# Comparative genome analyses of *Mycobacterium avium* reveal genomic features of its subspecies and strains that cause progression of pulmonary disease

**DOI:** 10.1038/srep39750

**Published:** 2017-01-03

**Authors:** Kei-ichi Uchiya, Shuta Tomida, Taku Nakagawa, Shoki Asahi, Toshiaki Nikai, Kenji Ogawa

**Affiliations:** 1 Department of Microbiology, Faculty of Pharmacy, Meijo University, Nagoya 468-8503, Japan; 2 Department of Biobank, Graduate School of Medicine, Dentistry and Pharmaceutical Sciences, Okayama University, Okayama 700-8558, Japan; 3 Department of Clinical Research, National Hospital Organization, Higashinagoya National Hospital, Nagoya 465-8620, Japan; 4 Department of Respiratory Medicine, National Hospital Organization, Higashinagoya National Hospital, Nagoya 465-8620, Japan

## Abstract

Pulmonary disease caused by nontuberculous mycobacteria (NTM) is increasing worldwide. *Mycobacterium avium* is the most clinically significant NTM species in humans and animals, and comprises four subspecies: *M. avium* subsp. *avium* (MAA), *M. avium* subsp. *silvaticum* (MAS), *M. avium* subsp. *paratuberculosis* (MAP), and *M. avium* subsp. *hominissuis* (MAH). To improve our understanding of the genetic landscape and diversity of *M. avium* and its role in disease, we performed a comparative genome analysis of 79 *M. avium* strains. Our analysis demonstrated that MAH is an open pan-genome species. Phylogenetic analysis based on single nucleotide variants showed that MAH had the highest degree of sequence variability among the subspecies, and MAH strains isolated in Japan and those isolated abroad possessed distinct phylogenetic features. Furthermore, MAP strains, MAS and MAA strains isolated from birds, and many MAH strains that cause the progression of pulmonary disease were grouped in each specific cluster. Comparative genome analysis revealed the presence of genetic elements specific to each lineage, which are thought to be acquired via horizontal gene transfer during the evolutionary process, and identified potential genetic determinants accounting for the pathogenic and host range characteristics of *M. avium*.

Whole-genome sequencing and comparative genome analysis of a large number of related strains have recently emerged as a cost-effective and convenient approach for addressing many microbiological questions, such as evolution, outbreaks, antibiotic resistance, and pathogenicity. This approach has been applied to several pathogens, such as *Staphylococcus aureus*^1^, *Streptococcus pneumonias*^2^, *Vibrio cholera*^3^, and *Propionibacterium acnes*^4^,^5^.

Nontuberculous mycobacteria (NTM) are increasingly recognized as an important cause of morbidity in many countries, including the United States and Japan^6^,^7^,^8^,^9^,^10^. NTM infection is thought to be caused by NTM that reside in the environment, including in garden soil and bathrooms^11^,^12^,^13^. Pulmonary disease caused by NTM, which is both intractable and infectious, has variable clinical manifestations. Although some patients remain stable without treatment, others show signs of deterioration despite long-term multidrug therapy^9^,^14^,^15^. The causative NTM species of pulmonary disease vary by country. In Japan, the pulmonary disease-causing NTM with the highest incidence is *Mycobacterium avium*, followed by *M. intracellulare*, *M. kansasii*, and *M. abscessus*; today, the incidence per 100,000 population is estimated to be 14.7^8^.

Among NTM species, *M. avium* is the most clinically significant species in humans and animals and comprises four subspecies that have specific pathogenic and host range characteristics as follows: *M. avium* subsp. *avium* (MAA) and *M. avium* subsp. *silvaticum* (MAS) are avian pathogens; *M. avium* subsp. *paratuberculosis* (MAP) causes John’s disease in ruminants; and *M. avium* subsp. *hominissuis* (MAH) infects mainly pigs and humans^16^,^17^,^18^. MAH is the causative pathogen of two main types of disease in humans: disseminated disease in immunocompromised hosts such as individuals infected with human immunodeficiency virus (HIV), and pulmonary disease in individuals without systemic immunosuppression^9^. MAA and MAH have also been isolated from other animals, such as deer, cattle, and horses^19^. MAP is transmitted into herds via the fecal-oral route through pastures or water contaminated with the feces of infected animals^18^. In contrast, MAS infects wood pigeons almost exclusively and is taxonomically very close to MAA^17^. However, the genetic differences among the four subspecies are still unknown.

The mechanisms involved in the development and exacerbation of pulmonary NTM disease have yet to be elucidated, but are possibly the result of both host and bacterial factors. Maekura *et al*. reported that pulmonary disease patients with serotype 4 MAH strains have significantly poorer prognoses than those with other serotypes^20^. In recent studies, variable number tandem repeats (VNTR) typing analysis of isolates from patients with pulmonary NTM disease revealed that isolates from patients with progressive pulmonary disease and those with stable pulmonary disease are clustered differently^21^,^22^,^23^, which suggests the involvement of bacterial factors in the progression of pulmonary NTM disease.

In our previous study, we determined the complete genome sequence of strain TH135 isolated from a serious case with worsening pulmonary MAH disease^24^, and further demonstrated the presence of a circular plasmid, pMAH135^25^. This novel plasmid consists of 194,711 nucleotides and has 164 coding sequences (CDSs), some of which encode proteins involved in the pathogenicity of mycobacteria and their resistance to antimicrobial agents. The screening of MAH isolates from humans and pigs for genes located on pMAH135 revealed that these genes are more commonly detected in isolates from patients with pulmonary MAH disease than in HIV-positive patients. However, the genes are almost entirely absent in isolates from pigs, suggesting that pMAH135 influences not only the pathological manifestations of MAH disease, but also the host specificity.

In this study, to improve our understanding of the genetic landscape and diversity of *M. avium* and its role in disease, we performed a comparative genome analysis of 79 *M. avium* strains including 46 novel MAH genomes.

## Results

### *M. avium* strains and general genomic features

To investigate the genomic features of MAH that cause the progression of pulmonary disease, we sequenced the genomes of 46 MAH isolates from 17 patients with progressive disease and 29 patients with stable disease, in addition to the previously determined complete genome sequence of MAH strain TH135^24^, which was used as a reference genome in the analysis. The average genome size of 46 novel MAH isolates was 5.351 Mb (ranging from 4.981 to 5.895 Mb), and the G + C content was 68.9% (ranging from 68.5 to 69.3%), with 370 contigs on average (ranging from 162 to 1,062) (Table 1). The relationship between genome size and G + C content was examined in these strains (Supplementary Fig. S1). Interestingly, a negative correlation (correlation coefficient −0.9491; p < 0.0001), wherein the G + C content became smaller as the genome size became larger. This indicates that MAH strains with a large genome might have evolved through acquisition of exogenous genes via phages, transposons, or other integrative and conjugative elements.

Our analysis included 32 additional *M. avium* genomes that are publicly available (Table 1). These genomes include all *M. avium* subspecies: fifteen MAH strains, six MAA strains, seven MAP strains, one MAS strain, and three *M. avium* strains of unknown subspecies.

### Phylogenetic relationships among *M. avium* genomes

**Figure 1:**
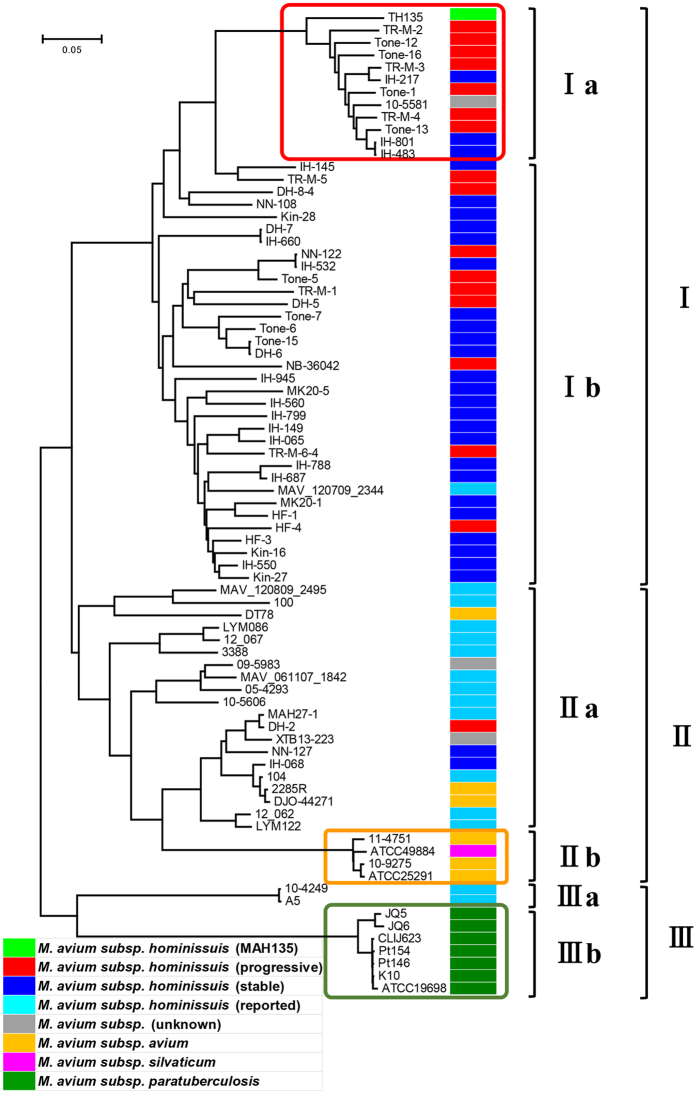
Phylogenetic tree of 79 *M. avium* strains using 101,139 SNVs on 3,404,650 bases of core genome regions. The evolutionary history was inferred using the neighbor-joining method^60^. The evolutionary distances were computed using the Maximum Composite Likelihood method^61^ and represent the number of base substitutions per site. Evolutionary analyses were conducted in MEGA6^58^. The analysis included nucleotide sequences of genomes from 79 *M. avium* strains (red, MAH isolates from progressive disease patients; blue, MAH isolates from stable disease patients; light blue, MAH strains reported publicly; gray, other *M. avium* strains; orange, MAA strains; pink, MAS strain; green, MAP strains; and light green, MAH reference strain TH135).

Comparative genome analysis of the 79 *M. avium* strains including four subspecies revealed that the total length of the core region, which was shared by all the 79 *M. avium* isolates, was 3,404,650 bp with 101,139 unique single nucleotide variants (SNVs). Phylogenetic analysis based on the SNVs in the core regions showed that the *M. avium* strains were roughly classified into three clusters: cluster I, cluster II, and cluster III (Fig. 1). Furthermore, it was shown that each cluster has a distinctive subcluster (cluster Ia, cluster IIb or cluster IIIb) comprised of strains with genetic distances that are clearly different from those of the others. Cluster I contained 93.5% (43/46) of the MAH genome sequenced in this study, MAH strain TH135 (reference strain), MAV_120709_2344, and strain 105581 of unknown subspecies. Cluster II contained 12 MAH strains isolated abroad, 2 strains of unknown subspecies, 3 MAH strains (IH-065, NN-127, and DH-2) sequenced in this study, all MAA strains, and MAS strain ATCC49884. Thus, the MAH strains isolated in Japan and those isolated in the United States, Belgium, and Germany formed different clusters. In cluster II, MAA strains were divided largely into two subclusters: cluster IIb containing 3 of the 6 MAA strains along with MAS strain ATCC49884 and cluster IIa containing the rest of the MAA strains along with MAH strains. Interestingly, all strains in cluster IIb were of avian origin. Among cluster III, cluster IIIb contained all MAP strains, and cluster IIIa contained two MAH strains.

MAH infects both pigs and humans, and several studies in Europe using molecular genotyping methods have shown high genetic similarity between MAH strains isolated from humans and pigs^26^,^27^,^28^,^29^, indicating the possibility of a common source of MAH infection among humans and pigs, as well as the possibility of pig–human zoonotic infections. In this study, three MAH isolates (10–5606, LYM122, and LYM086) from pigs in the United States and Belgium belonged to cluster IIa, which contains MAH human isolates, without the formation of a specific cluster. In addition, no major genetic differences between human and pig isolates were found. Specifically, high genetic similarity was found between human (12_062 and 12_067) and pig (LYM122 and LYM086) isolates in Belgium^30^, suggesting either a common source of infection or the zoonotic potential of MAH. However, this problem needs to be examined in detail using genome sequences of many pig isolates.

Next, we examined the phylogenetic relationships among 46 MAH isolates from patients with either progressive or stable disease. Of the 46 isolates, 43 (93.5%) were grouped in cluster I, while only 3 were in cluster II (Fig. 1). It is worth mentioning that 41.2% (7/17) of isolates from the patients with progressive disease and 10.3% (3/29) of those from the patients with stable disease were in cluster Ia, a subcluster of cluster I with a distinctively different genetic distance. The ratio of isolates from patients with progressive disease to those from patients from stable disease was significantly higher in cluster Ia than in other subclusters (Supplementary Table S1, p = 0.025 by Fisher’s exact test). It would be interesting to clarify the pathogenesis or evolutionary process of strain 105581, isolated in the United States, as it belongs in cluster Ia. These results indicate a specific genotype of MAH is associated with the progression of pulmonary MAH disease.

**Figure 2:**
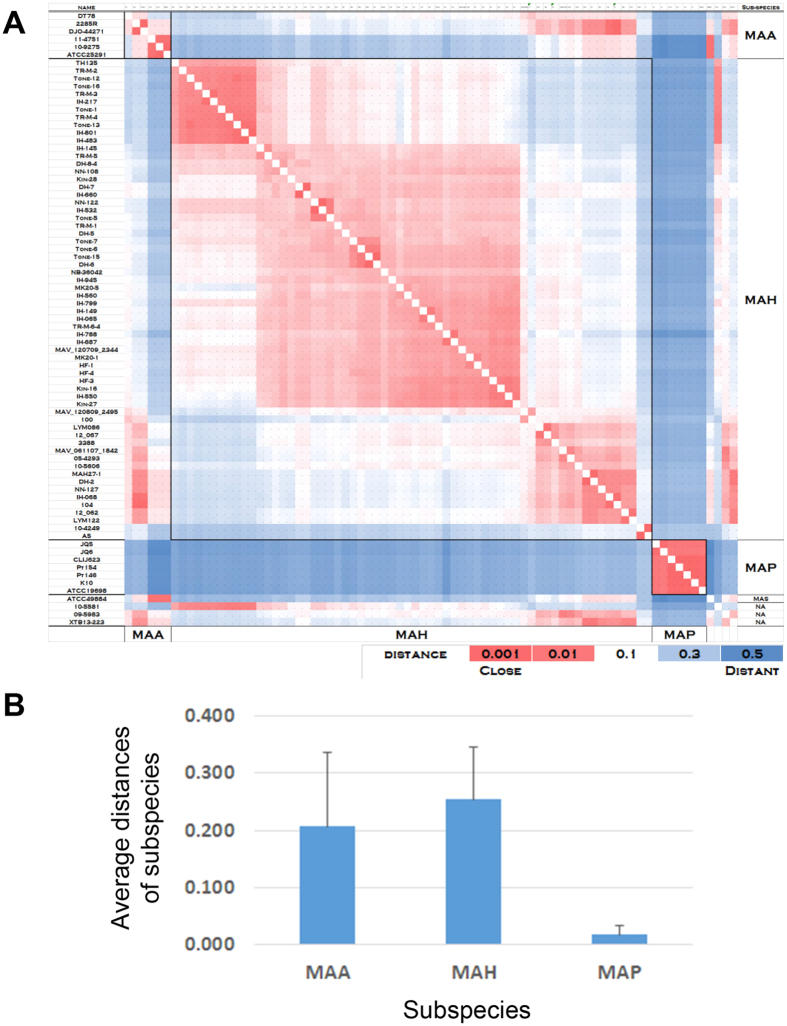
Distance matrix among 79 *M. avium* isolates. (**A**) Distances between 79 *M. avium* isolates were calculated as nucleotide substitution rates at all 101,139 SNV sites, and are colored according to the scale bar. (**B**) Average distance was calculated for MAAs, MAHs, and MAPs.

Furthermore, phylogenetic analysis currently shows that the degree of sequence diversity of *M. avium* genomes is different between subspecies. Therefore, we calculated the distance (substitution rate at the 101,139 SNV sites in the core regions) between each pair of *M. avium* subspecies (Fig. 2A). The average distance of MAH strains was 0.254, while those of MAA and MAP strains were 0.207 and 0.018, respectively (Fig. 2B). This result indicates that, among the *M. avium* subspecies, MAH exhibits the highest degree of sequence diversity, whereas the least diversity is observed in MAP.

### SNV distribution and conserved region of *M. avium* genomes

**Figure 3:**
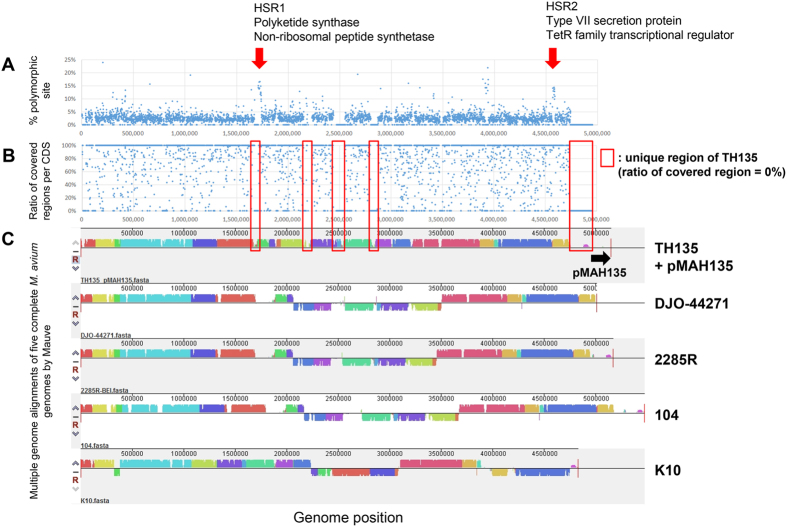
SNV distribution in the core regions. (**A**) SNV frequencies (percentages of polymorphic sites) of each coding sequence (CDS) in core regions. (**B**) Ratio of covered regions of each CDS. (**C**) Multiple genome alignments of five complete *M. avium* genomes (strains TH135 with pMAH135, DJO-44271, 2285 R, 104, and K10) analyzed using Mauve software.

To understand whether there are “hot spot regions (HSRs)” for mutation and/or recombination in the *M. avium* genomes, we calculated the percentage of polymorphic sites for each CDS (Fig. 3A). We found several HSRs in *M. avium* genome, such as HSR1 (ranging from 1,712,045 to 1,737,933) encoding CDSs involved in mycobactin biosynthesis, including several nonribosomal peptide synthetases and polyketide synthases, and HSR2 (ranging from 4,565,477 to 4,587,364) encoding type VII secretion proteins and TetR family transcriptional regulators (Table 2). Mycobacteria synthesize siderophores, named mycobactin, to capture iron, which is an essential nutrient for almost all organisms^31^. HSR1 contains 7 CDSs with sequence homologies to the MbtB–MbtH proteins of other mycobacteria, and is involved in the synthesis of the siderophore core of mycobactin^31^. Pathogenic mycobacteria carry the type VII secretion systems, namely the ESX systems (ESX-1 to ESX-5), which are responsible for secreting 6-kDa early secreted antigenic target (ESAT-6) or mycobacteria-specific proteins with conserved N-terminal domains containing prolyl-glutamic acid (PE) and prolyl-prolyl glutamic acid (PPE) motifs^32^,^33^. Thus, CDSs associated with the pathogenicity of *M. avium* were present in HSR1 and HSR2. In addition, a lower ratio of covered regions per CDS corresponded to a lower similarity region analyzed by comparing five complete genomes, such as MAH strain TH135, MAA strain DJO-44271, MAA strain 2285 R, MAH strain 104, and MAP strain K10, using MAUVE. This result showed that the non-core regions identified in this analysis were unique or specific to each isolate (Fig. 3B,C).

### Noncore genome regions in cluster Ia

**Figure 4:**
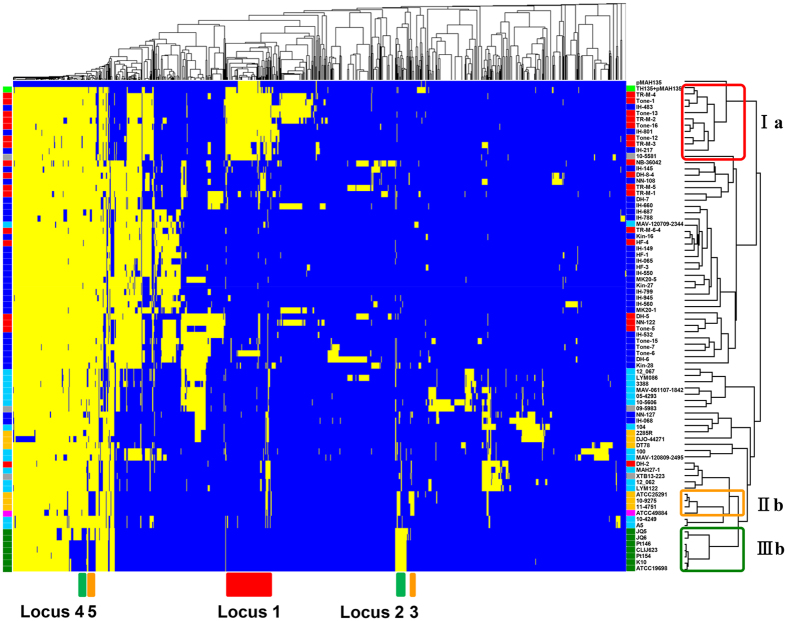
Noncore genome comparison of 79 *M. avium* strains. Rows represent 79 *M. avium* genomes and the sequence of the pMAH135 plasmid from strain TH135, and columns represent 1187 noncore regions longer than 1000 bp. Genomes and noncore regions were clustered based on similarity. The presence of a noncore region is shown in yellow, and its absence is shown in blue. Locus 1 is mostly unique to cluster Ia, which consists of many isolates from progressive disease patients. The presence of pMAH135 is also characteristic of cluster Ia. Loci 2 and 3 are mostly unique to cluster IIIb that includes MAP strains, and cluster IIb that includes MAA and MAS strains, respectively.

By comparing the genome sequences of the 79 *M. avium* strains, including the sequences of the chromosome and pMAH135 from strain TH135 as references, we identified noncore genome regions that were not shared by all strains (Fig. 4). The total length of the noncore regions was approximately 7.86 Mb, meaning each strain has on average about 100 kb unique sequences. It is noteworthy that the mean G + C content of 7.86 Mb noncore regions is 65.4%, which is much lower than that of the 79 *M. avium* genomes (68.9%). This result suggests that part of the specific noncore regions might have originated from other species via horizontal gene transfer. Clustering analysis of 1187 noncore regions with more than 1000 bp showed that MAH isolates from the progressive disease patients were significantly grouped in a specific cluster (cluster Ia), which was completely consistent with the results of clustering analysis based on SNVs in the core regions (Fig. 4). Among the noncore regions, we identified a genomic region (locus 1) that was mostly specific to cluster Ia.

Locus 1 in the specific noncore regions contains CDSs encoded by strain TH135 chromosome, the pMAH135 plasmid, and genomes of other mycobacteria except for strain TH135 (Fig. 4 and Supplementary Table S2). Specific regions (SR)-2, SR-4, SR-7, SR-8, and SR-9, which were previously identified on strain TH135 chromosome^24^, were present on this locus. These regions have low G + C content compared with the mean G + C content of strain TH135 chromosome and are flanked by genes that encode integrases of phage origin and/or transposases derived from transposons, which is an additional sign of foreign origin. Among these regions, SR-2 carries virulence-associated genes, namely, *mce* family genes and *mmpL* gene (Supplementary Table S2). Mycobacteria have several *mce* operons that comprise two *yrbE* and six *mce* genes (*mceA* to *mceF*), which are homologous to the permeases and substrate-binding proteins of ABC transporters, respectively^34^. MmpL and MmpS proteins are reported to mediate the transport of lipid metabolites for the biosynthesis of cell wall lipids in mycobacteria^35^,^36^,^37^. The high content of lipids, such as mycolic acids, in the cell walls plays a pivotal role in host survival^38^. Locus 1 also contains CDSs encoded by pMAH135. This plasmid encodes 164 CDSs, some of which encode proteins involved in mycobactin biosynthesis and the type VII secretion system, associated with pathogenicity of mycobacteria (Supplementary Table S2). Furthermore, locus 1 contains CDS with a 100% sequence identity to MmpL protein of *M. avium* 10–5581. These results suggest that virulence-associated CDSs in the noncore region specific to cluster Ia, probably acquired by horizontal gene transfer during evolution, play an important role in the pathogenicity of MAH isolates from patients with progressive disease.

### Plasmid analysis of MAH isolates

**Figure 5:**
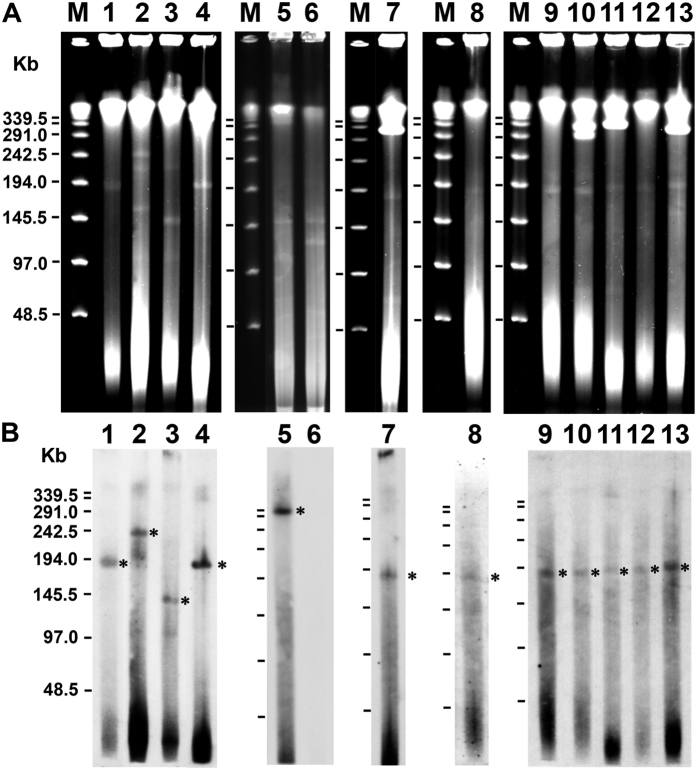
Analysis of plasmids from MAH isolates. PFGE of S1 nuclease-digested total DNA of MAH isolates (**A**) and Southern hybridization with a probe derived from MAH_p01 (**B**). Asterisks show plasmid bands hybridized with the probe. Lanes 1–13 represent, in order, strains TH135, IH-217, TR-M-3, TR-M-2, Tone-6, TR-M-1, IH-483, Tone-12, IH-801, Tone-13, Tone-1, Tone-16, and TR-M-4. The molecular size of the lambda ladder PFG marker (lane M) is shown in the left panel.

As shown in Fig. 4, CDSs encoded by pMAH135 were present in 12 strains (Tone-6, TR-M-1, IH-217, TR-M-3, IH-483, IH-801, Tone-12, Tone-13, Tone-1, Tone-16, TR-M-4, and TR-M-2) from 46 MAH isolates, suggesting the presence of pMAH135. Except for strains Tone-6 and TR-M-1 in cluster Ib, 10 strains were grouped in cluster Ia that consisted of many isolates from progressive disease patients (Fig. 1). We therefore conducted plasmid analysis of these strains with S1-PFGE and Southern hybridization, using a pMAH135-specific probe (MAH_p01). Among these 12 strains, 8 (IH-483, IH-801, Tone-12, Tone-13, Tone-1, Tone-16, TR-M-4, and TR-M-2) belonged to cluster Ia and carried a plasmid of approximately 194-kb, which was similar in size to pMAH135 (Fig. 5A) and MAH_p01 (Fig. 5B). The three remaining strains—Tone-6, IH-217, and TR-M-3—had MAH_p01 located on plasmids of approximately 388 kb, 242 kb, and 145 kb in size, respectively. Because MAH_p01 was absent, strain TR-M-1 was analyzed using MAH_p47 as another pMAH135-specific probe, which showed that MAH_p47 was located on the chromosome, not the plasmid.

### Noncore genome regions in clusters IIb and IIIb

As shown in Fig. 4, we found noncore regions specific to clusters IIb and IIIb. Locus 2 was present in cluster IIIb that contains MAP strains. Locus 3 was found in cluster IIb that contains MAS and MAA strains isolated from birds. These loci contain CDSs with sequence homologies to integrases and/or transposases, with lower G + C content compared with the mean G + C content of 79 *M. avium* genomes (Supplementary Table S2). Furthermore, locus 2 carries virulence-related CDSs with sequence homologies to genes encoding Mec, MmpL/MmpS, and PPE proteins, while CDS showing sequence homologies to genes encoding PPE protein is present in locus 3. PE/PPE family proteins are recognized as virulence factors that participate in antigenic variation and host immune evasion^39^. On the other hand, some regions were missing from the strains found in clusters IIb and IIIb. Locus 4 was absent from cluster IIIb strains, and locus 5 was absent from clusters IIb and IIIb strains. These loci contain several CDSs with sequence homologies to PPE proteins. Although the roles of many of the CDSs in the regions specific to clusters IIb and IIIb or in the missing regions are unclear, it is thought that these CDSs affects the specific pathogenic and host range characteristics of strains in clusters IIb and IIIb. Further study is necessary to elucidate the functions of those isolate-specific CDSs.

### *M. avium* pan-genome

**Figure 6:**
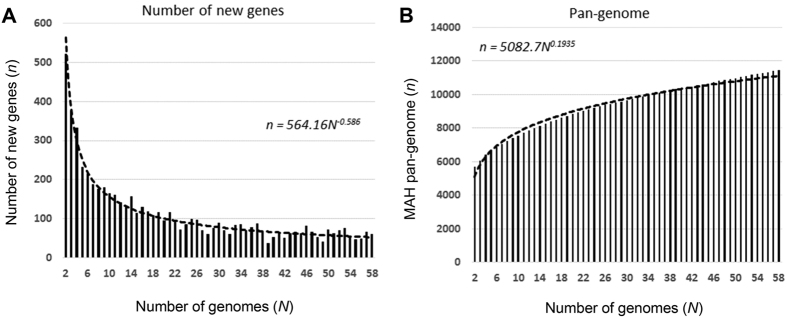
*M. avium* pan-genome. (**A**) Power law regression for new genes (*n*) discovered on the addition of new genome sequences (*N*). (**B**) Power law regression for total genes (*n*) accumulated with the addition of new genome sequences (*N*). Circles show the medians of *n* for 200 simulations. Error bars indicate the standard deviations for the 200 simulations.

Based on the suggested diversity of MAH genome as described above, we estimated the pan-genome based on the 58 MAH genomes, including the reference strain TH135 genome and 11 additional publicly available MAH genomes. We first estimated the number of new genes that would be discovered by sequencing additional MAH genomes via power law regression analysis, *n* = *κN*^*–α*^ 40 (Fig. 6A). Our analysis identified α as 0.586. When the 58^th^ genome was added, the average number of new genes added by a novel genome was 52. We then estimated the number of MAH pan-genes that would be accumulated by sequencing additional MAH genomes using power law regression analysis, *n* = *κN*^*γ*^ (Fig. 6B). The exponent γ as 0.194, and MAH had 11,151 pan-genes (n = 58). Based on these results, the pan-genome of MAH is defined as open because the exponent α was less than one and γ was greater than zero^40^. These results indicate that MAH has a high degree of genomic diversity.

## Discussion

In our previous study, VNTR typing analysis using *M. avium* tandem repeats (MATR) of isolates from patients with pulmonary MAH disease demonstrated a relationship between VNTR genotype and disease progression. Furthermore, screening of these isolates for six genes located in pMAH135 indicated a relationship between disease progression and the presence of pMAH135 genes^23^. In this study, by comparing the genome sequences of 79 *M. avium* strains comprising four subspecies, we analyzed the phylogenetic relationships based on the SNVs among the *M. avium* subspecies, including MAH isolates from patients with different clinical courses, characterized the genetic diversity and features of SNVs in *M. avium* genomes, revealed the presence of genetic elements specific to each lineage phylogenetically classified into unique clusters, and identified potential genetic determinants associated with the host range characteristics of *M. avium,* as well as the progression of pulmonary MAH disease.

The phylogenetic analysis based on the SNVs in the core regions showed that the *M. avium* strains were roughly classified into three clusters. Furthermore, it was shown that each cluster has a distinctive subcluster (cluster Ia, cluster IIb or cluster IIIb), the constituent strains of which appear to have evolved from the common ancestor through unique evolutionary pathways (Fig. 1). MAH strains were present in clusters I through III, whereas all MAP strains belonged to cluster IIIb, and cluster IIb was formed specifically by MAA strains of avian origin and MAS strain. This suggests that strains in cluster IIIb have genomic feature associated with John’s disease and that MAA and MAS strains share genomic features that enable them to infect birds. Using VNTR analysis, Iwamoto *et al*. and Ichikawa *et al*. demonstrated a geographical difference in the genetic diversity of MAH^41^,^42^. In agreement with this, we found that MAH strains isolated in Japan formed a cluster (cluster I) that differs from the cluster (cluster II) containing MAH strains isolated in the United States or Germany, indicating that they have different genomic features. This may be one of the reasons for the high incidence of pulmonary MAH disease in Japan^8^.

The average distances of each *M. avium* subspecies calculated based on all SNV sites indicated that MAH strains have the highest degree of sequence diversity (Fig. 2). This is consistent with previous results of SNV-based multilocus sequencing analysis using 10 housekeeping genes^43^. Moreover, this analysis revealed that MAP strains exhibit the lowest sequence diversity. Our study further demonstrated that MAH is an open pan-genome species (Fig. 6), indicating that the acquisition and deletion of genetic elements occurred at high rates during the evolutionary process. Taken together, these results show that MAH is an *M. avium* species with high genetic diversity.

In our recent studies and that of Kikuchi *et al*., MATR-VNTR analysis of isolates from patients with pulmonary MAH disease demonstrated that isolates from progressive disease cases are grouped in a specific cluster^22^,^23^, and further revealed that many of the isolates from both groups are classified into the same cluster. These findings suggest that strains in this cluster are highly virulent. In this study, SNV-based phylogenetic analysis showed that isolates from progressive disease patients were notably grouped in cluster Ia (p = 0.025) (Fig. 1 and Supplementary Table S1). Interestingly, isolates in cluster Ia fully corresponded with those in the specific cluster described above obtained by MATR-VNTR analysis examining an identical set of isolates^23^. Although other clusters did not exhibit a complete match, these results indicate that genotypes based on SNVs overlap with VNTR genotypes. Taken together, these results suggest that the isolates in cluster Ia have unique genomic features associated with the progression of pulmonary MAH disease, and demonstrate that MATR-VNTR analysis can distinguish isolates from progressive disease patients simply. Therefore, this analysis is a clinically useful approach.

By analyzing the noncore regions, we identified genomic element (locus 1) specific to cluster Ia consisting of many MAH isolates from progressive disease patients (Fig. 4). This genomic element harbors virulence genes that account for the progression of pulmonary MAH disease. On locus 1, SR-2, which was previously identified as one of the specific regions on strain TH135 chromosome^24^, is present and carries virulence-associated *mce* family genes and *mmpL* gene. Although the precise mechanisms of Mce proteins remain unclear, they are thought to be mainly involved in the entry of mycobacteria into mammalian cells and their subsequent survival^44^,^45^. Furthermore, locus 1 contains CDSs that are encoded on pMAH135 and involved in mycobactin biosynthesis and the type VII secretion system. De Voss *et al*. reported that a *M. tuberculosis* mutant lacking the *mbtB* gene interrupts the biosynthesis of mycobactin and impairs the growth of macrophages^46^, suggesting that mycobactin plays a significant role in the pathogenicity of mycobacteria. ESX-5, which is similar to the ESX-related proteins encoded on pMAH135, mediates the secretion of ESAT-6-like proteins EsxN and EsxP, and is involved in inducing cell death in infected macrophages and modulating the immune response^47^. Thus, pMAH135 is thought to be involved in MAH pathogenicity. Interestingly, Ummels *et al*. reported that pMAH135 is a conjugative plasmid in slow-growing mycobacteria species, including *M. avium*^48^. Plasmid analysis by S1-PFGE revealed that eight isolates carry pMAH135 (Fig. 5), which probably originated from other mycobacteria by conjugation.

We previously reported that five Is*Mav6* genes, which is a novel insertion sequence, are coded by strain TH135 chromosome^24^,^49^. One was inserted into Shine–Dalgarno region of the *cfp29* gene^50^, which is involved in the induction of interferon–γ production in hosts with mycobacterial infection, thus suggesting its influence on MAH pathogenicity. Interestingly, the frequency of isolates with Is*Mav6* inserted into the *cfp29* gene determined by analyzing an identical set of isolates—but not those with Is*Mav6—*was significantly higher in patients with progressive disease than in those with stable disease^51^. Comparisons of the detection rates between Is*Mav6* inserted into the *cfp29* gene and four potential virulence factors specific to cluster Ia strains in isolates from patients with progressive disease showed that the former was higher than the latter (Supplementary Table S3). These results suggest that Is*Mav6* inserted into the *cfp29* gene is one factor related to the progression of pulmonary MAH disease. Taken together, cluster Ia strains acquired genetic regions (e.g. SR-2 and pMAH135) encoding virulence genes via horizontal transfer during the evolutionary process, thereby acquiring pathogenicity resulting in disease progression. It will be intriguing in the future to discover how such virulence factors are involved in pathogenicity. Cluster Ia also contained three strains isolated from the stable disease patients. In addition, some isolates from the progressive disease patients did not belong to cluster Ia, but caused disease progression despite having identical genomic characteristics to isolates from the stable disease patients. Such cases could be explained by our hypothesis that suggests the influence of host factors, such as the host immune system, are stronger than bacterial factors on the clinical course of pulmonary MAH disease.

One of the clinical problems in the treatment of pulmonary NTM disease is the difficulty in judging the appropriate time to start therapy. The clinical course of patients with pulmonary NTM disease is diverse; some patients are stable without any treatment and others have worsening symptoms despite drug therapy, which leads to severe lung damage^9^,^14^. Clarithromycin-based multidrug therapy is recommended for pulmonary MAH disease; however, it requires a long treatment period (18–24 months) and is associated with the risk of adverse reactions from the use of multiple drugs, which places considerable financial, psychological, and physical burdens on patients^52^,^53^,^54^. Furthermore, the timing of treatment initiation influences the outcome and is therefore also important. Clear criteria for determining the timing of treatment are not currently available. The findings of this study demonstrated the potential of virulence genes encoded by SR-2 and pMAH135 specific to isolates from progressive disease patients as one indicator of the need to initiate therapy.

However, this study has some limitations. There were no objective standards used in judging patient status, either progressive or stable, and the classification of patients depended entirely on the decision made by each physician in charge at the individual participating hospitals. In addition, this was a retrospective study with a small number of subjects. Thus, this investigation can be regarded as a preliminary study. A future prospective study should evaluate our findings, and further investigate the way in which the virulence genes specific to isolates from progressive disease patients are involved in the pathogenicity.

In conclusion, the findings from our comparative genome analysis of 79 *M. avium* strains comprising four subspecies provided a perspective on the genetic diversity and evolution of *M. avium* strains, as well as genomic evidence that may explain the differences among *M. avium* subspecies. Of note, we revealed the presence of genetic elements specific to each lineage, which are thought to be acquired via horizontal gene transfer during the evolutionary process, and identified potential genetic determinants associated with not only the progression of pulmonary MAH disease but also the host range characteristics of *M. avium*. In the future, genome sequences of many NTM isolates should be investigated to elucidate in detail the various problems associated with NTM.

## Methods

### Bacterial strains

MAH strain TH135 isolated from the sputum of a seriously ill patient with worsening pulmonary MAH disease at Higashinagoya National Hospital of the National Hospital Organization was used as the reference strain. As reported previously^23^, 46 MAH isolates used in genome analysis in this study were provided by nine National Hospital Organization hospitals across Japan. These clinical isolates were obtained from the sputa of 46 patients with distinct clinical courses (see below). Only one strain per patient was analyzed in this study. Of the patients diagnosed with pulmonary MAH disease (corresponding to the diagnostic criteria of the American Thoracic Society and the Infectious Diseases Society of America^52^) between July 2008 and September 2009, those who started clarithromycin-based multidrug treatment within 18 months, based on decisions made by the corresponding physician-in-charge because of deterioration in the patients’ condition, were classed as the progressive disease group (n = 17). Those who did not receive treatment because their condition was stable were classed as the stable disease group (n = 29). During the observation period, the condition of each patient was evaluated several times a year based on chest radiograph findings (including chest computed tomographic images), clinical symptoms, and/or microbiological findings. Parameters of age, sex, type of pulmonary disease, and the presence of underlying disease were not significantly different between the two groups^23^.

### Identification of subspecies of *M. avium*, growth condition, and DNA isolation

The subspecies of *M. avium* clinical isolates was identified as MAH by sequence analysis of the 3′ fragment of the *hsp65* gene^55^. The organism was grown in Middlebrook 7H9 liquid medium supplemented with 10% oleic acid/albumin/dextrose/catalase enrichment (Difco Laboratories, Detroit, MI) at 37 °C. DNA was extracted using the illustra bacterial genomicPrep Mini Spin Kit (GE Healthcare, Buckinghamshire, UK) according to the manufacturer’s instructions.

### Whole genome sequencing and analysis of the core/noncore regions and pan-genome

All 46 MAH genomes were sequenced using Illumina MiSeq (150 bp, paired-end) with Nextera XT DNA Library Prep Kit (Illumina, CA) and assembled using CLC Genomics Workbench (Qiagen Inc., Valencia, CA) with the default settings. Core/noncore regions and pan-genome analysis were performed as previously described^5^. Briefly, the core regions were defined as genome sequences present in all 79 genomes, while the noncore regions were defined as those not present in all genomes. MAH strain TH135 was used as the reference genome. All other 78 genome sequences were mapped to the reference genome using Nucmer^56^. The unique regions from each genome were identified and added to the reference genome until all unique regions from all genomes were included in the pan-genome. Core regions were then subtracted from the pan-genome and the remaining regions were defined as noncore regions. Protein coding sequences were predicted by GeneMark^57^ using MAH strain 104 as a reference. The 79 concatenated sequences of the 101,139 single nucleotide variant (SNV) nucleotides in the core regions were used to construct a phylogenetic tree of the *M. avium* genomes. MEGA6^58^ was used to calculate the distance based on the 101,139 SNVs in the core regions. Comparative genomic analysis was performed with five complete *M. avium* genomes (strains TH135 with pMAH135, DJO-44271, 2285 R, 104, and K10) using the Mauve multiple genome aligner^59^.

### Plasmid analysis

Plasmid DNA analysis was performed using S1-pulsed-field gel electrophoresis (PFGE) and Southern hybridization using a specific probe, as described previously^25^. Bacteria in agarose gel plugs were treated with lysozyme and proteinase K, before digestion of the total DNA in the plugs by 10 U S1 nuclease (Takara Bio, Shiga, Japan) for 10 min at 37 °C. PFGE was performed using the Bio-Rad CHEF-DR III system at 14 °C and 6 V/cm^2^ for 24 h with a switch time of 1.6–21.3 s. After electrophoresis, Southern hybridization analysis including probe labeling was performed using the DIG High Prime DNA Labeling and Detection Starter Kit II (Roche, Mannheim, Germany) according to the manufacturer’s instructions. A pMAH135-specific probe was prepared by PCR with specific primers for MAH_p01, which encodes the *repA* gene (thought to be the origin of replication for pMAH135), and DNA from MAH strain TH135.

### Statistical analysis

The correlation between genome size and G + C content was analyzed using Spearman’s rank correlation. Fisher’s exact test was used for categorical variables. All statistical analysis was performed using GraphPad Prism version 5.0 (GraphPad Software, San Diego, CA). *P* values < 0.05 were considered significant.

### Ethics Statement

This study was approved and carried out in accordance with guidelines and regulations by the Ethics Review Committee for Human Research of the Higashinagoya National Hospital, and written informed consent was obtained from all patients.

### Nucleotide sequence accession numbers

Draft genome sequences reported here were deposited in DDBJ/EMBL/GenBank under accession no. PRJDB502.

## Additional Information

**How to cite this article**: Uchiya, K. *et al*. Comparative genome analyses of *Mycobacterium avium* reveal genomic features of its subspecies and strains that cause progression of pulmonary disease. *Sci. Rep.* **7**, 39750; doi: 10.1038/srep39750 (2017).

**Publisher's note:** Springer Nature remains neutral with regard to jurisdictional claims in published maps and institutional affiliations.

## Supplementary Material

Supplementary Information File #1

## Figures and Tables

**Table 1:** General feature of 75 *M. avium* genomes.

Genome no.	Strain	Subspecies^a^	Source	Cluster^b^	Genome size (Mb)	GC (%)	No. of contigs
1	DH-6	MAH	stable disease patient^c^	Ib	5.895	68.5	537
2	DH-7	MAH	stable disease patient	Ib	5.077	69.2	276
3	HF-1	MAH	stable disease patient	Ib	5.252	68.9	313
4	HF-3	MAH	stable disease patient	Ib	5.258	68.9	399
5	IH-065	MAH	stable disease patient	Ib	5.122	69.1	484
6	IH-068	MAH	stable disease patient	IIa	5.215	69.1	287
7	IH-145	MAH	stable disease patient	Ib	5.218	69.0	342
8	IH-149	MAH	stable disease patient	Ib	5.211	69.1	366
9	IH-217	MAH	stable disease patient	Ia	5.331	69.1	162
10	IH-483	MAH	stable disease patient	Ia	5.753	68.7	415
11	IH-550	MAH	stable disease patient	Ib	5.192	69.1	419
12	IH-532	MAH	stable disease patient	Ib	5.440	68.9	327
13	IH-560	MAH	stable disease patient	Ib	5.384	68.9	328
14	IH-660	MAH	stable disease patient	Ib	5.359	68.9	552
15	IH-687	MAH	stable disease patient	Ib	5.090	69.1	272
16	IH-788	MAH	stable disease patient	Ib	5.397	68.6	1,062
17	IH-799	MAH	stable disease patient	Ib	5.106	69.1	402
18	IH-801	MAH	stable disease patient	Ia	5.335	69.0	376
19	IH-945	MAH	stable disease patient	Ib	5.090	69.1	510
20	Kin-16	MAH	stable disease patient	Ib	5.217	69.0	268
21	Kin-27	MAH	stable disease patient	Ib	5.127	69.1	347
22	Kin-28	MAH	stable disease patient	Ib	5.390	68.9	327
23	MK20-1	MAH	stable disease patient	Ib	5.453	68.8	486
24	MK20-5	MAH	stable disease patient	Ib	5.340	69.0	295
25	NN-108	MAH	stable disease patient	Ib	5.250	69.0	416
26	NN-127	MAH	stable disease patient	IIa	5.209	69.1	242
27	Tone-6	MAH	stable disease patient	Ib	5.812	68.6	434
28	Tone-7	MAH	stable disease patient	Ib	5.463	68.9	377
29	Tone-15	MAH	stable disease patient	Ib	5.498	68.8	511
30	DH-2	MAH	progressive disease patient	IIa	4.981	69.3	308
31	DH-5	MAH	progressive disease patient	Ib	5.449	68.9	326
32	DH-8-4	MAH	progressive disease patient	Ib	5.130	69.1	389
33	HF-4	MAH	progressive disease patient	Ib	5.222	69.0	304
34	NB-36042	MAH	progressive disease patient	Ib	5.482	68.8	382
35	NN-122	MAH	progressive disease patient	Ib	5.751	68.7	367
36	Tone-1	MAH	progressive disease patient	Ia	5.659	68.7	371
37	Tone-5	MAH	progressive disease patient	Ib	5.232	69.1	262
38	Tone-12	MAH	progressive disease patient	Ia	5.398	68.9	354
39	Tone-13	MAH	progressive disease patient	Ia	5.742	68.7	295
40	Tone-16	MAH	progressive disease patient	Ia	5.354	69.0	226
41	TR-M-1	MAH	progressive disease patient	Ib	5.442	68.8	336
42	TR-M-2	MAH	progressive disease patient	Ia	5.515	68.8	299
43	TR-M-3	MAH	progressive disease patient	Ia	5.280	69.0	352
44	TR-M-4	MAH	progressive disease patient	Ia	5.588	68.8	294
45	TR-M-5	MAH	progressive disease patient	Ib	5.264	69.0	286
46	TR-M-6-4	MAH	progressive disease patient	Ib	5.156	69.1	322
47	TH135^d^	MAH	progressive disease patient	Ia	4.951	69.3	1
48	MAH27-1	MAH	household dust (Germany)	IIa	4.794	69.0	977
49	12_062	MAH	human (Belgium)	IIa	5.095	69.0	175
50	12_067	MAH	human (Belgium)	IIa	5.108	69.0	216
51	LYM122	MAH	pig (Belgium)	IIa	5.093	69.0	175
52	LYM086	MAH	pig (Belgium)	IIa	5.308	69.0	199
53	10-5606	MAH	pig (USA)	IIa	5.529	68.7	1,690
54	10-4249	MAH	deer (USA)	IIIa	4.787	69.2	924
55	05-4293	MAH	bird (USA)	IIa	5.156	69.2	190
56	3388	MAH	unknown (USA)	IIa	5.052	69.2	128
57	100	MAH	unknown (USA)	IIa	5.499	68.8	340
58	A5	MAH	unknown (USA)	IIIa	4.871	69.4	50
59	104	MAH	AIDS patient (USA)	IIa	5.475	69.0	1
60	MAV_061107_1842	MAH	lung disease patient (USA)	IIa	5.321	69.1	16
61	MAV_120809_2495	MAH	lung disease patient (USA)	IIa	5.712	68.9	97
62	MAV_120709_2344	MAH	lung disease patient (USA)	Ib	5.629	68.8	62
63	10–5581	unknown	elephant (USA)	Ia	5.330	69.0	325
64	09–5983	unknown	whitetail deer (USA)	IIa	5.167	68.9	1,113
65	XTB13-223	unknown	human (USA)	IIa	5.130	69.2	33
66	10-9275	MAA	redtail hawk (USA)	IIb	4.795	69.1	886
67	11–4751	MAA	ruddy duck (USA)	IIb	4.835	69.2	577
68	2285 R	MAA	human (USA)	IIa	5.169	69.1	1
69	DJO-44271	MAA	human (USA)	IIa	5.011	69.1	1
70	DT78	MAA	water buffalo’s ileum (USA)	IIa	4.960	69.2	1,201
71	ATCC25291	MAA	hen (Canada)	IIb	4.858	69.2	258
72	ATCC49884	MAS	unknown	IIb	4.713	69.2	808
73	K10	MAP	Johne’s disease cow (USA)	IIIb	4.830	69.3	1
74	ATCC19698	MAP	cow (USA)	IIIb	4.736	69.3	1,092
75	Pt146	MAP	unknown (Australia)	IIIb	4.583	69.0	955
76	Pt154	MAP	unknown (Australia)	IIIb	4.500	68.9	1,077
77	CLIJ623	MAP	unknown (Australia)	IIIb	4.523	69.0	915
78	JQ5	MAP	unknown (USA)	IIIb	4.735	69.1	176
79	JQ6	MAP	unknown (USA)	IIIb	4.697	69.0	176

^a^MAH; *M. avium* subsp. *hominissuis*, MAA; *M. avium* subsp. *avium*, MAS; *M. avium* subsp. *silvaticum*, MAP; *M. avium* subsp. *paratuberculosis*.

^b^Cluster was classified by the phylogenetic analysis as shown in Fig. 1.

^c^Patients with different clinical courses in pulmonary diseases described in Material and Methods.

^d^Strain TH135 was used as the reference strain.

**Table 2:** List of CDSs with higher percentage of polymorphic site.

Region^a^	Location (position)	Length	Covered length	Ratio of covered region per CDS	Polymorphic site (%)	Locus tag	Description
HSR1	1712045–1713700	1,656	1,012	61%	14%	MAH_RS08025	2,3-dihydroxybenzoate-AMP ligase
HSR1	1713807–1717304	3,498	3,028	87%	15%	MAH_RS08030	non-ribosomal peptide synthetase
HSR1	1717313–1718077	765	765	100%	14%	MAH_RS08035	thioesterase
HSR1	1718083–1719402	1,320	1,320	100%	14%	MAH_RS08040	polyketide synthase
HSR1	1719402–1722443	3,042	1,275	42%	16%	MAH_RS08045	polyketide synthase
HSR1	1722445–1729017	6,573	6,419	98%	14%	MAH_RS08050	non-ribosomal peptide synthetase
HSR1	1729014–1733456	4,443	3,896	88%	17%	MAH_RS08055	non-ribosomal peptide synthetase
HSR1	1733453–1734739	1,287	1,287	100%	12%	MAH_RS08060	lysine 6-monooxygenase
HSR1	1734720–1734935	216	216	100%	13%	MAH_RS08065	protein mbtH
HSR1	1735111–1735647	537	537	100%	12%	MAH_RS08070	hypothetical protein
HSR1	1735941–1736456	516	516	100%	12%	MAH_RS08075	low molecular weight antigen MTB12
HSR1	1736450–1737340	891	656	74%	15%	MAH_RS08080	RNA polymerase sigma-70 factor
HSR1	1737337–1737933	597	597	100%	12%	MAH_RS08085	4-carboxymuconolactone decarboxylase
HSR2	4565477–4567111	1,635	1,635	100%	14%	MAH_RS21020	type VII secretion protein EccB
HSR2	4567108–4568961	1,854	1,714	92%	10%	MAH_RS21025	type VII secretion AAA-ATPase EccA
HSR2	4570937–4571842	906	906	100%	13%	MAH_RS21035	SAM-dependent methyltransferase
HSR2	4577124–4578050	927	927	100%	12%	MAH_RS21070	hypothetical protein
HSR2	4578152–4578814	663	647	98%	14%	MAH_RS21075	TetR family transcriptional regulator
HSR2	4580166–4581641	1,476	1,415	96%	14%	MAH_RS21085	acyltransferase
HSR2	4582027–4582575	549	549	100%	14%	MAH_RS21090	hypothetical protein
HSR2	4583277–4583933	657	657	100%	13%	MAH_RS21100	TetR family transcriptional regulator
HSR2	4583966–4585072	1,107	1,107	100%	13%	MAH_RS21105	alpha/beta hydrolase
HSR2	4585163–4587364	2,202	2,202	100%	11%	MAH_RS21110	acyl-CoA dehydrogenase

^a^Regions HSR1 and HSR2 are shown in Fig. 3.
