# Supplementary material for: Comparative genome analyses of Mycobacterium avium reveal genomic features of its subspecies and strains that cause progression of pulmonary disease
Source: Sci Rep. 2017 Jan 3;7:39750. doi: 10.1038/srep39750 (PMC5206733; doi:10.1038/srep39750)
Supplement: Supplementary Information File #1 [file srep39750-s1.pdf]

Supplementary Information

Comparative genome analyses of *Mycobacterium avium* reveal genomic features of its subspecies and strains that cause progression of pulmonary disease

Kei-ichi Uchiya[1,†,*], Shuta Tomida[2,†], Taku Nakagawa[3,4], Shoki Asahi[1], Toshiaki Nikai[1], and Kenji Ogawa[3,4]

[1]Department of Microbiology, Faculty of Pharmacy, Meijo University, Nagoya 468-8503, Japan

[2]Department of Biobank, Graduate School of Medicine, Dentistry and Pharmaceutical Sciences, Okayama University, Okayama 700-8558, Japan

[3]Department of Clinical Research, National Hospital Organization, Higashinagoya National Hospital, Nagoya 468-8620, Japan

[4]Department of Respiratory Medicine, National Hospital Organization, Higashinagoya National Hospital, Nagoya 468-8620, Japan

†These authors contributed equally to this work.

* Correspondence should be addressed to K.U. (kuchiya@meijo-u.ac.jp)

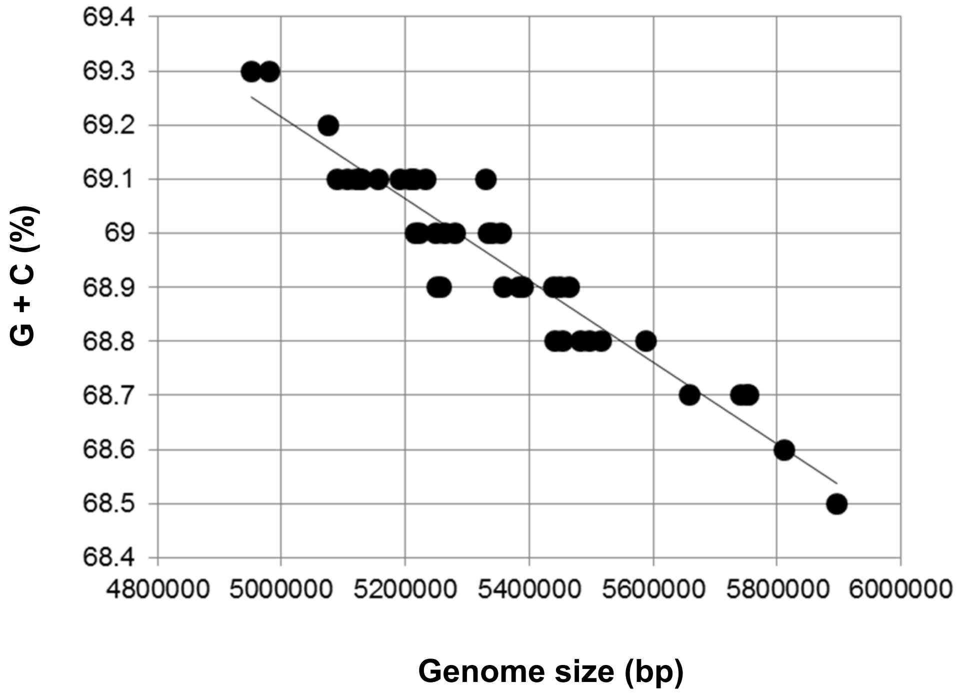

Supplementary Figure 1. Relationships between genome size and G+C content from 46 MAH isolates.

The correlation was analyzed using a Spearman's rank correlation, which was strongly negative (correlation coefficient –0.9491; $p < 0.0001$).

Supplementary Table 1. Relationships between SNV-based genotype and disease progression in pulmonary NTM disease.

Cluster[a]	Progressive disease group (n = 17)	Stable disease group (n = 29)	*P* value[b]
Ia	7 (41.2%)	3 (10.3%)	0.025
Ib + IIa	10 (58.8%)	26 (89.7%)	

Cluster	Progressive disease group (n = 17)	Stable disease group (n = 29)	*P* value
Ia	7 (41.2%)	3 (10.3%)	0.042
Ib	9 (52.9%)	24 (82.8%)	
IIa	1 (5.9%)	2 (6.9%)	

[a] Each cluster was classified by the phylogenetic analysis as shown in Fig. 1.

[b] *P* values were calculated using Fisher's exact test.

Supplementary Table S2. List of CDSs encoded on each locus.

Locus	Locus_tag	Predicted product	Species with most similar sequence	Amino acid sequence identity (%)
**Locus 1**				
	MAH_0359	hypothetical protein	M. avium subsp. hominissuis TH135	100
	MAH_0684	hypothetical protein	M. avium subsp. hominissuis TH135	99
	MAH_0685	hypothetical protein	M. avium subsp. hominissuis TH135	100
SR-2	MAH_0778	MMPL family protein	M. avium subsp. hominissuis TH135	100
	MAH_0779	diacylglycerol O-acyltransferase	M. avium subsp. hominissuis TH135	100
	MAH_0780	cytochrome P450	M. avium subsp. hominissuis TH135	100
	MAH_0781	luciferase family protein	M. avium subsp. hominissuis TH135	100
	MAH_0782	L-carnitine dehydratase/bile acid-inducible protein F	M. avium subsp. hominissuis TH135	100
	MAH_0783	thioesterase	M. avium subsp. hominissuis TH135	99
	MAH_0784	cytochrome P450	M. avium subsp. hominissuis TH135	99
	MAH_0785	ubiquinone biosynthesis protein UbiE	M. avium subsp. hominissuis TH135	100
	MAH_0786	cytochrome P450	M. avium subsp. hominissuis TH135	100
	MAH_0787	short chain dehydrogenase	M. avium subsp. hominissuis TH135	99
	MAH_0793	hypothetical protein	M. avium subsp. hominissuis TH135	100
	MAH_0794	ABC transporter permease	Mycobacterium marinum M	96
	MAH_0795	ABC transporter permease	M. avium subsp. hominissuis TH135	99
	MAH_0796	virulence factor Mce family protein	M. avium subsp. hominissuis TH135	100
	MAH_0797	virulence factor Mce family protein	M. avium subsp. hominissuis TH135	100
	MAH_0798	virulence factor Mce family protein	M. avium subsp. hominissuis TH135	100
	MAH_0799	virulence factor Mce family protein	M. avium subsp. hominissuis TH135	99
	MAH_0800	virulence factor Mce family protein	M. avium subsp. hominissuis TH135	100
	MAH_0801	virulence factor Mce family protein	M. avium subsp. hominissuis TH135	100
	MAH_0803	hypothetical protein	M. avium subsp. hominissuis TH135	100
	MAH_0804	hypothetical protein	M. avium subsp. hominissuis TH135	100
	MAH_0805	hypothetical protein	M. avium subsp. hominissuis TH135	100
	MAH_0806	hypothetical protein	M. avium subsp. hominissuis TH135	100
	MAH_0807	hypothetical protein	M. avium subsp. hominissuis TH135	100
	MAH_0808	transcriptional regulatory protein	M. avium subsp. hominissuis TH135	100
	MAH_0814	TetR family transcriptional regulator	M. avium subsp. hominissuis TH135	100
	MAH_0815	enoyl-CoA hydratase	M. avium subsp. hominissuis TH135	100
	MAH_0816	hypothetical protein	M. avium subsp. hominissuis TH135	100
	MAH_0818	long-chain-fatty-acid--CoA ligase	M. avium subsp. hominissuis TH135	100
	MAH_0819	membrane protein	M. avium subsp. hominissuis TH135	100
	MAH_0820	hypothetical protein	M. avium subsp. hominissuis TH135	99
SR-4	MAH_1773	DNA (cytosine-5-)-methyltransferase	M. avium subsp. hominissuis TH135	100
	MAH_1775	adenine specific DNA methylase	M. avium subsp. hominissuis TH135	100
	MAH_1776	Predicted ATPase, AAA+ superfamily	M. avium subsp. hominissuis TH135	100
	MAH_1780	hypothetical protein	M. avium subsp. hominissuis TH135	100
	MAH_1781	hypothetical protein	M. avium subsp. hominissuis TH135	99
	MAH_1782	hypothetical protein	M. avium subsp. hominissuis TH135	99
	MAH_1783	hypothetical protein	M. avium subsp. hominissuis TH135	100
	MAH_2772	hypothetical protein	M. avium subsp. hominissuis TH135	100
	MAH_2773	hypothetical protein	M. avium subsp. hominissuis TH135	99
	MAH_2774	hypothetical protein	M. avium subsp. hominissuis TH135	100
	MAH_2775	hypothetical protein	M. avium subsp. hominissuis TH135	100
SR-7	MAH_2921	endonuclease	M. avium subsp. hominissuis TH135	99
	MAH_2922	hypothetical protein	M. avium subsp. hominissuis TH135	100
	MAH_2923	hypothetical protein	M. avium subsp. hominissuis TH135	99
	MAH_2926	hypothetical protein	M. avium subsp. hominissuis TH135	99
	MAH_2928	probable phirv2 prophage integrase	M. avium subsp. hominissuis TH135	100
	MAH_2929	hypothetical protein	M. avium subsp. hominissuis TH135	100
	MAH_2930	hypothetical protein	M. avium subsp. hominissuis TH135	100
	MAH_2931	hypothetical protein	M. avium subsp. hominissuis TH135	100
	MAH_2932	hypothetical protein	M. avium subsp. hominissuis TH135	100
	MAH_2933	hypothetical protein	M. avium subsp. hominissuis TH135	99
	MAH_2934	hypothetical protein	M. avium subsp. hominissuis TH135	100
	MAH_2935	hypothetical protein	M. avium subsp. hominissuis TH135	100
	MAH_2936	hypothetical protein	M. avium subsp. hominissuis TH135	100
	MAH_2939	hypothetical protein	M. avium subsp. hominissuis TH135	100
	MAH_2940	hypothetical protein	M. avium subsp. hominissuis TH135	99
SR-8	MAH_3052	hypothetical protein	M. avium subsp. hominissuis TH135	100
	MAH_3053	hypothetical protein	M. avium subsp. hominissuis TH135	100
	MAH_3054	HsdR family type I site-specific deoxyribonuclease	M. avium subsp. hominissuis TH135	100
	MAH_3054	HsdR family type I site-specific deoxyribonuclease	M. avium subsp. hominissuis TH135	100
	MAH_3055	hypothetical protein	M. avium subsp. hominissuis TH135	100
	MAH_3056	Methylase_S	M. avium subsp. hominissuis TH135	100
	MAH_3057	type I restriction/modification system DNA methylase hsdM	M. avium subsp. hominissuis TH135	100
	MAH_3058	hypothetical protein	M. avium subsp. hominissuis TH135	100
	MAH_3060	Fis family transcriptional regulator	M. avium subsp. hominissuis TH135	99
	MAH_0055	hypothetical protein	M. avium subsp. hominissuis TH135	100
	MAH_1380	AraC family transcriptional regulator	M. avium subsp. hominissuis TH135	100
	MAH_1381	thiJ/PfpI domain-containing protein	M. avium subsp. hominissuis TH135	100
	MAH_1595	3-(3-hydroxyphenyl)propionate hydroxylase	M. avium subsp. hominissuis TH135	99
	MAH_1596	enoyl-CoA hydratase	M. avium subsp. hominissuis TH135	100
	MAH_1597	acyl-CoA dehydrogenase domain-containing protein	M. avium subsp. hominissuis TH135	100
	MAH_1598	pyruvate dehydrogenase E1 component	M. avium subsp. hominissuis TH135	100
	MAH_1599	biphenyl-2,3-diol 1,2-dioxygenase	M. avium subsp. hominissuis TH135	100
	MAH_1600	fumarylacetoacetase	M. avium subsp. hominissuis TH135	100
	MAH_1713	TetR family transcriptional regulator	M. avium subsp. hominissuis TH135	99

	MAH_1714	short-chain dehydrogenase/reductase	M. avium subsp. hominissuis TH135	100
	MAH_1883	transposase	M. avium subsp. hominissuis TH135	100
	MAH_1883	transposase	M. avium subsp. hominissuis TH135	100
	MAH_2252	membrane protein	M. avium subsp. hominissuis TH135	100
	MAH_2253	Bacterial regulatory proteins, tetR family	M. avium subsp. hominissuis TH135	100
	MAH_2323	transposase	M. avium subsp. hominissuis TH135	100
	MAH_2383	transposase IS4 family protein	M. avium subsp. hominissuis TH135	100
	MAH_2491	oxidoreductase, 2-nitropropane dioxygenase	M. avium subsp. hominissuis TH135	99
	MAH_2492	malonate semialdehyde decarboxylase	M. avium subsp. hominissuis TH135	100
	MAH_2493	LysR family transcriptional regulator	M. avium subsp. hominissuis TH135	100
	MAH_2494	TetR family transcriptional regulator	M. avium subsp. hominissuis TH135	100
	MAH_2495	phytoene dehydrogenase-like oxidoreductase	M. avium subsp. hominissuis TH135	100
SR-9	MAH_3202	DNA segregation ATPase FtsK/SpoIIIE-like protein	M. avium subsp. hominissuis TH135	100
	MAH_3203	regulatory protein	M. avium subsp. hominissuis TH135	99
	MAH_3205	hypothetical protein	M. avium subsp. hominissuis TH135	100
	MAH_3206	creatinine amidohydrolase	M. avium subsp. hominissuis TH135	100
	MAH_3207	integral membrane protein	M. avium subsp. hominissuis TH135	100
	MAH_3208	flavodoxin-like protein	M. avium subsp. hominissuis TH135	99
	MAH_3209	ECF subfamily RNA polymerase sigma-24 factor	M. avium subsp. hominissuis TH135	100
	MAH_3210	putative zinc-finger	M. avium subsp. hominissuis TH135	99
	MAH_3211	membrane protein	M. avium subsp. hominissuis TH135	100
	MAH_3212	TetR family transcriptional regulator	M. avium subsp. hominissuis TH135	100
	MAH_3213	haloalkane dehalogenase	M. avium subsp. hominissuis TH135	99
	MAH_3214	hypothetical protein	M. avium subsp. hominissuis TH135	100
	MAH_3215	glutamate-cysteine ligase GshA	M. avium subsp. hominissuis TH135	100
	MAH_3216	dimethylhistidine N-methyltransferase	M. avium subsp. hominissuis TH135	100
	MAH_3217	haloacid dehalogenase	M. avium subsp. hominissuis TH135	100
	MAH_3218	haloacid dehalogenase	M. avium subsp. hominissuis TH135	100
	MAH_3219	response regulator receiver domain-containing protein	M. avium subsp. hominissuis TH135	99
	MAH_3220	alkylhydroperoxidase	M. avium subsp. hominissuis TH135	100
	MAH_3221	hypothetical protein	M. avium subsp. hominissuis TH135	100
	MAH_3222	ketosteroid isomerase-related protein	M. avium subsp. hominissuis TH135	100
	MAH_3223	alpha/beta hydrolase	M. avium subsp. hominissuis TH135	100
	MAH_3230	haloalkane dehalogenase	M. avium subsp. hominissuis TH135	100
	MAH_3231	alpha/beta hydrolase family	M. avium subsp. hominissuis TH135	100
	MAH_3232	hypothetical protein	M. avium subsp. hominissuis TH135	100
	MAH_3233	hypothetical protein	M. avium subsp. hominissuis TH135	99
	MAH_p01	replication protein Rep	M. avium subsp. hominissuis TH135	100
	MAH_p02	Transcription factor WhiB	M. avium subsp. hominissuis TH135	100
	MAH_p03	hypothetical protein	M. avium subsp. hominissuis TH135	100
	MAH_p04	soj protein	M. avium subsp. hominissuis TH135	100
	MAH_p05	peptide transporter	M. avium subsp. hominissuis TH135	100
	MAH_p06	peptide transporter	M. avium subsp. hominissuis TH135	100
	MAH_p07	plasmid stability protein	M. avium subsp. hominissuis TH135	99
	MAH_p08	PilT domain-containing protein/plasmid stability protein StbE	M. avium subsp. hominissuis TH135	100
	MAH_p09	hypothetical protein	M. avium subsp. hominissuis TH135	99
	MAH_p10	hypothetical protein	M. avium subsp. hominissuis TH135	98
	MAH_p11	hypothetical protein	M. avium subsp. hominissuis TH135	100
	MAH_p12	hypothetical protein	M. avium subsp. hominissuis TH135	100
	MAH_p13	protein of unknown function DUF2384	M. avium subsp. hominissuis TH135	100
	MAH_p14	hypothetical protein	M. avium subsp. hominissuis TH135	100
	MAH_p15	YbaB/EbfC DNA-binding family	M. avium subsp. hominissuis TH135	99
	MAH_p16	PPE family	M. avium subsp. hominissuis TH135	100
	MAH_p17	Na+/proline symporter	M. avium subsp. hominissuis TH135	100
	MAH_p18	hypothetical protein	M. avium subsp. hominissuis TH135	100
	MAH_p19	hypothetical protein	M. avium subsp. hominissuis TH135	100
	MAH_p20	hypothetical protein	M. avium subsp. hominissuis TH135	100
	MAH_p20	hypothetical protein	M. avium subsp. hominissuis TH135	100
	MAH_p21	hypothetical protein	M. avium subsp. hominissuis TH135	100
	MAH_p22	hypothetical protein	M. avium subsp. hominissuis TH135	100
	MAH_p23	twitching motility protein PilT	M. avium subsp. hominissuis TH135	99
	MAH_p24	prevent-host-death family protein	M. avium subsp. hominissuis TH135	100
	MAH_p25	breaking-rejoining enzymes, C-terminal catalytic domain	M. avium subsp. hominissuis TH135	100
	MAH_p26	NADH-quinone oxidoreductase subunit N	M. avium subsp. hominissuis TH135	99
	MAH_p27	oxidoreductase	M. avium subsp. hominissuis TH135	99
	MAH_p28	NADH dehydrogenase	M. avium subsp. hominissuis TH135	100
	MAH_p29	NADH-ubiquinone oxidoreductase	M. avium subsp. hominissuis TH135	100
	MAH_p30	NADH:ubiquinone oxidoreductase subunit 6 (chain J)	M. avium subsp. hominissuis TH135	100
	MAH_p31	NADH dehydrogenase subunit 1	M. avium subsp. hominissuis TH135	100
	MAH_p32	hypothetical protein	M. avium subsp. hominissuis TH135	100
	MAH_p33	NADH-ubiquinone/plastoquinone oxidoreductase chain 3	M. avium subsp. hominissuis TH135	100
	MAH_p37	heavy metal transport/detoxification protein	M. avium subsp. hominissuis TH135	100
	MAH_p38	heavy metal translocating P-type ATPase	M. avium subsp. hominissuis TH135	100
	MAH_p39	MerR family transcriptional regulator	M. avium subsp. hominissuis TH135	100
	MAH_p41	alpha/beta hydrolase	M. avium subsp. hominissuis TH135	100
	MAH_p43	polyketide synthase MbtD	M. avium subsp. hominissuis TH135	100
	MAH_p44	polyketide synthase MbtC	M. avium subsp. hominissuis TH135	99
	MAH_p45	thioesterase	M. avium subsp. hominissuis TH135	100
	MAH_p46	acetyltransferase	M. avium subsp. hominissuis TH135	100
	MAH_p47	non-ribosomal peptide synthetase MbtE	M. avium subsp. hominissuis TH135	100
	MAH_p48	non-ribosomal peptide synthetase MbtF	M. avium subsp. hominissuis TH135	99
	MAH_p49	phenyloxazoline synthase MbtB	M. avium subsp. hominissuis TH135	100
	MAH_p50	dehydrogenase	M. avium subsp. hominissuis TH135	99
	MAH_p51	EsaT-6 like protein EsxN	M. avium subsp. hominissuis TH135	100
	MAH_p52	EsaT-6 like protein EsxP	M. avium subsp. hominissuis TH135	100
	MAH_p53	PPE family protein	M. avium subsp. hominissuis TH135	100

MAH_p54	PE family protein	M. avium subsp. hominissuis TH135	100
MAH_p55	amino acid adenylation enzyme/thioester reductase family prote	M. avium subsp. hominissuis TH135	100
MAH_4588	TetR family transcriptional regulator	M. avium subsp. hominissuis TH135	100
MAH_4589	hypothetical protein	M. avium subsp. hominissuis TH135	99
MAH_p91	PPE family protein	M. avium subsp. hominissuis TH135	100
MAH_p92	hypothetical protein	M. avium subsp. hominissuis TH135	100
MAH_p93	mRNA interferase PemK	M. avium subsp. hominissuis TH135	100
MAH_p94	phosphotyrosine protein phosphatase	M. avium subsp. hominissuis TH135	100
MAH_p95	arsenic-transport integral membrane protein ArsC	M. avium subsp. hominissuis TH135	100
MAH_p96	bleomycin resistance protein	M. avium subsp. hominissuis TH135	100
MAH_p97	ArsR family transcriptional regulator	M. avium subsp. hominissuis TH135	100
MAH_p98	hypothetical protein	M. avium subsp. hominissuis TH135	100
MAH_p98	hypothetical protein	M. avium subsp. hominissuis TH135	100
MAH_p101	N-acetylglutamate synthase	M. avium subsp. hominissuis TH135	100
MAH_p106	hypothetical protein	M. avium subsp. hominissuis TH135	100
MAH_p107	hypothetical protein	M. avium subsp. hominissuis TH135	100
MAH_p117	hypothetical protein	M. avium subsp. hominissuis TH135	100
MAH_p121	Transposase IS891/IS1136/IS1341 family	M. avium subsp. hominissuis TH135	99
MAH_p122	Methyl-accepting chemotaxis protein	M. avium subsp. hominissuis TH135	100
MAH_p123	Outer membrane receptor proteins, mostly Fe transport	M. avium subsp. hominissuis TH135	100
MAH_p124	hypothetical protein	M. avium subsp. hominissuis TH135	99
MAH_p126	hypothetical protein	M. avium subsp. hominissuis TH135	98
MAH_p127	hypothetical protein	M. avium subsp. hominissuis TH135	100
MAH_p128	hypothetical protein	M. avium subsp. hominissuis TH135	100
MAH_p129	Type IV secretory pathway VirD4 family protein	M. avium subsp. hominissuis TH135	100
MAH_p140	hypothetical protein	M. avium subsp. hominissuis TH135	100
MAH_p141	hypothetical protein	M. avium subsp. hominissuis TH135	100
MAH_p142	type VII secretion AAA-ATPase EccA	M. avium subsp. hominissuis TH135	100
MAH_p143	type VII secretion protein EccE	M. avium subsp. hominissuis TH135	100
MAH_p144	type VII secretion-associated serine protease	M. avium subsp. hominissuis TH135	100
MAH_p145	type VII secretion integral membrane protein EccD	M. avium subsp. hominissuis TH135	100
MAH_p146	hypothetical protein	M. avium subsp. hominissuis TH135	100
MAH_p147	hypothetical protein	M. avium subsp. hominissuis TH135	100
MAH_p148	EsaT-6 like protein EsxN	M. avium subsp. hominissuis TH135	100
MAH_p149	EsaT-6 like protein EsxP	M. avium subsp. hominissuis TH135	100
MAH_p150	PPE family protein	M. avium subsp. hominissuis TH135	100
MAH_p151	PE family protein	M. avium subsp. hominissuis TH135	100
MAH_p152	type VII secretion protein EccCa	M. avium subsp. hominissuis TH135	100
MAH_p153	type VII secretion protein EccB	M. avium subsp. hominissuis TH135	100
MAH_p154	NLP/P60 family protein	M. avium subsp. hominissuis TH135	100
MAH_p155	hypothetical protein	M. avium subsp. hominissuis TH135	99
MAH_p157	hypothetical protein	M. avium subsp. hominissuis TH135	100
MAH_p160	hypothetical protein	M. avium subsp. hominissuis TH135	100
MAH_p161	hypothetical protein	M. avium subsp. hominissuis TH135	100
MAH_p162	XRE family transcriptional regulator	M. avium subsp. hominissuis TH135	100
MAH_p163	Transcription factor WhiB family protein	M. avium subsp. hominissuis TH135	100
MAH_p164	hypothetical protein	M. avium subsp. hominissuis TH135	100
MAH_p58	linear gramicidin synthetase subunit D	M. avium subsp. hominissuis TH135	100
MAH_p59	integral membrane drug efflux protein	M. avium subsp. hominissuis TH135	100
MAH_p60	formyl transferase domain-containing protein	M. avium subsp. hominissuis TH135	100
MAH_p61	hypothetical protein	M. avium subsp. hominissuis TH135	98
MAH_p63	DUF305 domain-containing protein	M. avium subsp. hominissuis TH135	100
MAH_p66	erfK/YbiS/YcfS/YnhG family protein	M. avium subsp. hominissuis TH135	100
	regulated in copper repressor	M. avium subsp. hominissuis TH135	99
MAH_p73	metal cation transporting P-type ATPase ctpV	M. avium subsp. hominissuis TH135	100
MAH_p74	hypothetical protein	M. avium subsp. hominissuis TH135	100
MAH_p75	regulated in copper repressor	M. avium subsp. hominissuis TH135	100
MAH_p76	predicted metal-binding integral membrane protein	M. avium subsp. hominissuis TH135	100
MAH_p77	oxidase	M. avium subsp. hominissuis TH135	100
MAH_p78	copper-sensing transcriptional repressor csoR	M. avium subsp. hominissuis TH135	100
MAH_p81	pyridoxal-5'-phosphate-dependent protein subunit beta	M. avium subsp. hominissuis TH135	100
MAH_p82	putative zinc-finger	M. avium subsp. hominissuis TH135	100
MAH_p83	ornithine cyclodeaminase	M. avium subsp. hominissuis TH135	100
MAH_p84	Orn/DAP/Arg decarboxylase 2	M. avium subsp. hominissuis TH135	100
MAH_p85	MATE efflux family protein	M. avium subsp. hominissuis TH135	100
MAH_p86	DNA polymerase III subunits gamma	M. avium subsp. hominissuis TH135	100
MAH_p87	hypothetical protein W7U_10090	M. avium subsp. hominissuis TH135	100
MAH_p88	multidrug resistance protein MdtH	M. avium subsp. hominissuis TH135	100
	hypothetical protein	M. avium 10-5581	99
	hypothetical protein	M. avium 10-5581	100
	hypothetical protein	M. avium 10-5581	100
	hypothetical protein	M. avium 10-5581	100
	hypothetical protein	M. avium 10-5581	100
	hypothetical protein	M. avium 10-5581	100
	hypothetical protein	M. avium 10-5581	100
	hypothetical protein	M. avium 10-5581	97
	MmpL family protein	M. avium 10-5581	100
	transfer protein traSA	M. avium subsp. hominissuis	100
	prophage integrase	M. avium subsp. hominissuis	99
	hypothetical protein	M. avium 10-5581	100
	hypothetical protein	M. avium 10-5581	100
	addiction module protein	M. avium 10-5581	99
	antitoxin	M. avium 10-5581	100
	transglycosylase, partial	M. avium 10-5581	100
	plasmid replication initiator protein	M. gordonae	100
	plasmid replication initiator protein	M. gordonae	100

	serine recombinase	M. gordonae	99
	nlpC/P60 family protein	M. avium MAV_120709_2344	100
	fatty acid hydroxylase superfamily proteir	M. avium MAV_120709_2344	100
	bacterial regulatory helix-turn-helix s, AraC family proteir	M. avium MAV_120709_2344	99
	copper-translocating P-type ATPase	M. avium MAV_120709_2344	100
	hypothetical protein	M. avium MAV_120709_2344	100
	adenine nucleotide alpha hydrolases superfamily	M. avium MAV_120709_2344	100
	tatD related DNase family protein	M. avium MAV_120709_2344	99
	csbD family protein	M. avium MAV_120709_2344	100
	hypothetical protein	M. avium MAV_120709_2344	99
	aldehyde dehydrogenase family proteir	M. avium MAV_120709_2344	100
	oxidoreductase molybdopterin binding domain proteir	M. avium MAV_120709_2344	100
	putative conserved transmembrane protein	M. avium MAV_120709_2344	100
	hypothetical protein	M. avium MAV_120709_2344	100
	pyridine nucleotide-disulfide oxidoreductase family proteir	M. avium MAV_120709_2344	99
	hypothetical protein	M. avium MAV_120709_2344	100
	hypothetical protein	M. avium MAV_120709_2344	98
	RNA polymerase sigma24 factor	M. avium	100
	zinc-finger family protein	M. avium MAV_120709_2344	100
	haloalkane dehalogenase	M. avium MAV_120709_2344	100
	alkylhydroperoxidase AhpD family core domain proteir	M. avium MAV_120709_2344	100
	ATP dependent DNA ligase family protein	M. avium MAV_120709_2344	100
	histidine kinase-like ATPase domain protein	M. avium MAV_120709_2344	100
	hypothetical protein	M. avium MAV_120709_2344	100
	site-specific tyrosine recombinase XerC	M. avium subsp. hominissuis 100	100
	helix-turn-helix XRE-family like proteins	M. avium MAV_120709_2344	100
	hypothetical protein	Mycobacterium abscessus	100
	hypothetical protein	M. avium MAV_120709_2344	100
	hypothetical protein	M. avium subsp. hominissuis 100	100
	hypothetical protein	M. avium MAV_120709_2344	100
	putative sulfate-binding lipoprotein Subl	M. avium MAV_120709_2344	99
	glutathione S-transferase, C-terminal domain protein	M. avium MAV_120709_2344	100
	hypothetical protein	M. sp. UM_Kg17	69
	hypothetical protein	M. avium	76
	DNA methyltransferase	Mycobacterium sp. UNC280MFTsu5.1	86
	hypothetical protein	Mycobacterium	78
	sigma-54-dependent Fis family transcriptional regulator	Pelodictyon luteolum	68

Locus 2

MAP_4266	cyclase	M. avium subsp. paratuberculosis K-10	100
MAP_4268c	transcriptional regulator	M. avium subsp. paratuberculosis K-10	100
MAP_4269c	diaminopimelate decarboxylase	M. avium subsp. paratuberculosis K-10	99
MAP_0832c	transposase	M. avium subsp. paratuberculosis K-10	92
MAP_0095c	NADH-quinone oxidoreductase subunit I 1	M. avium subsp. paratuberculosis K-10	99
	hypothetical protein	Mycobacterium	100
MAP_0097c	fatty acid desaturase	M. avium subsp. paratuberculosis K-10	98
MAP_0098c	transcriptional regulator, TetR family	M. avium subsp. paratuberculosis K-10	100
MAP_0099	CsbD-like protein	M. avium subsp. paratuberculosis K-10	98
	hypothetical protein	M. vulneris	81
	carboxylate--amine ligase	M. avium	99
MAP_0102	hypothetical protein	M. avium subsp. paratuberculosis K-10	100
	hypothetical protein	M. avium subsp. paratuberculosis	100
	stromelysin-3	Lepisosteus oculatus	44
	hypothetical protein	M. avium subsp. paratuberculosis	100
MAP_0389	hypothetical protein	M. avium subsp. paratuberculosis K-10	99
MAP_0388	peroxidase	M. avium subsp. paratuberculosis K-10	99
MAP_0387	hypothetical protein	M. avium subsp. paratuberculosis K-10	100
	transport family protein	Mycobacterium	100
MAP_1824c	transcriptional regulator	M. avium subsp. paratuberculosis K-10	100
	hypothetical protein	*M. avium subsp. paratuberculosis 11-1786*	100
	hypothetical protein	M. avium subsp. paratuberculosis	99
MAP_0866	hypothetical protein	M. avium subsp. paratuberculosis K-10	100
	cell division protein FtsK, partial	*M. avium subsp. paratuberculosis 11-1786*	100
MAP_0864	hypothetical protein	M. avium subsp. paratuberculosis K-10	100
MAP_0861	hypothetical protein	M. avium subsp. paratuberculosis K-10	100
MAP_0859c	metallophosphatase	M. avium subsp. paratuberculosis K-10	100
MAP_0858	hypothetical protein	M. avium subsp. paratuberculosis K-10	100
MAP_0855	helicase	M. avium subsp. paratuberculosis K-10	100
MAP_0852	hypothetical protein	M. avium subsp. paratuberculosis K-10	100
MAP_0851	hypothetical protein	M. avium subsp. paratuberculosis K-10	100
	hypothetical protein	*M. avium subsp. paratuberculosis 11-1786*	100
MAP_0094	hypothetical protein	M. avium subsp. paratuberculosis K-10	100
MAP_2768c	integrase	M. avium subsp. paratuberculosis K-10	100
	site-specific recombinase XerD	*M. avium subsp. paratuberculosis S5*	100
MAP_2767c	hypothetical protein	M. avium subsp. paratuberculosis K-10	100
	DNA-binding protein	M. avium subsp. paratuberculosis	100
MAP_2765c	hypothetical protein	M. avium subsp. paratuberculosis K-10	100
MAP_2764c	hypothetical protein	M. avium subsp. paratuberculosis K-10	100
MAP_2763c	hypothetical protein	M. avium subsp. paratuberculosis K-10	100
MAP_2762c	hypothetical protein	M. avium subsp. paratuberculosis K-10	100
MAP_2761c	hypothetical protein	M. avium subsp. paratuberculosis K-10	100
MAP_2760c	hypothetical protein	M. avium subsp. paratuberculosis K-10	100
	hypothetical protein	M. avium subsp. paratuberculosis	99
	hypothetical protein	M. avium subsp. paratuberculosis	98
MAP_2759	hypothetical protein	M. avium subsp. paratuberculosis K-10	99

MAP_2758	hypothetical protein	M. avium subsp. paratuberculosis K-10	100
MAP_2757	hypothetical protein	M. avium subsp. paratuberculosis K-10	100
MAP_2756c	phage tail tape measure protein	M. avium subsp. paratuberculosis K-10	100
MAP_2755	hypothetical protein	M. avium subsp. paratuberculosis K-10	99
MAP_2754	CopG family transcriptional regulator	M. avium subsp. paratuberculosis K-10	100
MAP_2753	hypothetical protein	M. avium subsp. paratuberculosis K-10	100
MAP_2752	integrase	M. avium subsp. paratuberculosis K-10	100
MAP_2751	hypothetical protein	M. avium subsp. paratuberculosis K-10	100
MAP_3725	PPE family protein	M. avium subsp. paratuberculosis K-10	100
MAP_3726	Fe3+-siderophore ABC transporter permease	M. avium subsp. paratuberculosis K-10	100
MAP_3727	iron ABC transporter ATP-binding protein	M. avium subsp. paratuberculosis K-10	100
MAP_3728	Fe3+-citrate ABC transporter substrate-binding protein	M. avium subsp. paratuberculosis K-10	100
MAP_3729	taurine dioxygenase	M. avium subsp. paratuberculosis K-10	100
MAP_3730	methyltransferase	M. avium subsp. paratuberculosis K-10	100
MAP_3731c	cobalt ABC transporter	M. avium subsp. paratuberculosis K-10	100
MAP_3732c	ABC-type cobalt transport system, permease component	M. avium subsp. paratuberculosis K-10	100
MAP_3733c	membrane protein	M. avium subsp. paratuberculosis K-10	100
MAP_3734c	putative ABC transporter	M. avium subsp. paratuberculosis K-10	100
MAP_3735c	hemin ABC transporter ATP-binding protein	M. avium subsp. paratuberculosis K-10	100
MAP_3737	PPE family protein	M. avium subsp. paratuberculosis K-10	100
MAP_3738c	SAM-dependent methlyltransferase	M. avium subsp. paratuberculosis K-10	100
MAP_3739c	MFS transporter	M. avium subsp. paratuberculosis K-10	100
MAP_3740	thioester reductase	M. avium subsp. paratuberculosis K-10	100
MAP_2151	hypothetical protein	M. avium subsp. paratuberculosis K-10	99
	hypothetical protein	M. avium subsp. paratuberculosis	100
	hypothetical protein	M. avium subsp. paratuberculosis	98
MAP_2153	hypothetical protein	M. avium subsp. paratuberculosis K-10	100
	hypothetical protein	M. avium subsp. paratuberculosis	100
MAP_2154c	hypothetical protein	M. avium subsp. paratuberculosis K-10	100
MAP_3740	thioester reductase	M. avium subsp. paratuberculosis K-10	99
MAP_3741	hypothetical protein	M. avium subsp. paratuberculosis K-10	100
MAP_3742	thioester reductase	M. avium subsp. paratuberculosis K-10	99
MAP_3742	thioester reductase	M. avium subsp. paratuberculosis K-10	100
MAP_3743	hypothetical protein	M. avium subsp. paratuberculosis K-10	100
MAP_3744	thiazolinyl imide reductase	M. avium subsp. paratuberculosis K-10	100
MAP_3745	thioesterase	M. avium subsp. paratuberculosis K-10	100
MAP_3746	regulated in copper repressor	M. avium subsp. paratuberculosis K-10	100
MAP_3747c	putative cobalamin synthesis protein	M. avium subsp. paratuberculosis K-10	100
MAP_3749	3-ketoacyl-ACP reductase	M. avium subsp. paratuberculosis K-10	100
MAP_3750	membrane protein, MmpS family	M. avium subsp. paratuberculosis K-10	100
MAP_3752	acyl-CoA synthetase	M. avium subsp. paratuberculosis K-10	100
MAP_3753	hypothetical protein	M. avium subsp. paratuberculosis K-10	100
MAP_3755	hypothetical protein	M. avium subsp. paratuberculosis K-10	100
	ester cyclase	M. avium subsp. paratuberculosis K-10	99
MAP_3756c	N5,N10-methylene tetrahydromethanopterin reductase	M. avium subsp. paratuberculosis K-10	100
MAP_3757c	alpha/beta hydrolase	M. avium subsp. paratuberculosis K-10	100
MAP_3758c	Transcriptional regulator, AraC family	M. avium subsp. paratuberculosis K-10	100
MAP_2964c	phage integrase family protein	M. avium subsp. paratuberculosis K-10	100
MAP_2963c	Cro/CI family transcriptional regulator	M. avium subsp. paratuberculosis K-10	100
MAP_3765	PPE family protein	M. avium subsp. paratuberculosis K-10	100
MAP_3766	membrane protein	M. avium subsp. paratuberculosis K-10	100
	TIGR03943 family protein	M. avium subsp. paratuberculosis	100
MAP_3767c	30S ribosomal protein S18	M. avium subsp. paratuberculosis K-10	100
MAP_3768c	30S ribosomal protein S14	M. avium subsp. paratuberculosis K-10	100
MAP_3769c	50S ribosomal protein L33	M. avium subsp. paratuberculosis K-10	100
MAP_3770	putative cobalamin synthesis protein	M. avium subsp. paratuberculosis K-10	100
MAP_3771	50S ribosomal protein L31	M. avium subsp. paratuberculosis K-10	100
	transcription antitermination regulator	M. avium subsp. paratuberculosis	100
MAP_3772c	cobalamin biosynthesis protein CobW	M. avium subsp. paratuberculosis K-10	100
MAP_3773c	Fe2+/Zn2+ uptake regulation protein	M. avium subsp. paratuberculosis K-10	100
MAP_3774c	zinc ABC transporter permease	M. avium subsp. paratuberculosis K-10	100
MAP_3775c	ABC transporter, ATP-binding protein	M. avium subsp. paratuberculosis K-10	99
MAP_3776c	ABC transporter, periplasmic solute binding protein	M. avium subsp. paratuberculosis K-10	100
MAP_2085	transcriptional modulator of MazE/toxin MazF	M. avium subsp. paratuberculosis K-10	100
	hypothetical protein	M. avium subsp. paratuberculosis	100
MAP_3436c	hypothetical protein	M. avium subsp. paratuberculosis K-10	100
MAP_3437c	hypothetical protein	M. avium subsp. paratuberculosis K-10	100
	hypothetical protein	*Piloderma croceum F 1598*	41
MAP_2178	enterobactin synthase subunit E	M. avium subsp. paratuberculosis K-10	93
MAP_2179	hypothetical protein	M. avium subsp. paratuberculosis K-10	100
MAP_2180c	hypothetical protein	M. avium subsp. paratuberculosis K-10	100
MAP_2181c	TetR family transcriptional regulator	M. avium subsp. paratuberculosis K-10	100
MAP_2182c	nitroreductase	M. avium subsp. paratuberculosis K-10	100
MAP_2183c	cytochrome P450	M. avium subsp. paratuberculosis K-10	100
MAP_2184c	oxidoreductase	M. avium subsp. paratuberculosis K-10	100
MAP_2185c	amidohydrolase	M. avium subsp. paratuberculosis K-10	100
MAP_2186c	NAD-dependent aldehyde dehydrogenase	M. avium subsp. paratuberculosis K-10	99
MAP_2187c	NAD-dependent aldehyde dehydrogenase	M. avium subsp. paratuberculosis K-10	100
MAP_2188c	AMP-dependent synthetase	M. avium subsp. paratuberculosis K-10	99
MAP_2189	MCE-family protein	M. avium subsp. paratuberculosis K-10	100
MAP_2190	mammalian cell entry protein	M. avium subsp. paratuberculosis K-10	100
MAP_2191	mammalian cell entry protein	M. avium subsp. paratuberculosis K-10	99
MAP_2192	mammalian cell entry protein	M. avium subsp. paratuberculosis K-10	100
	mammalian cell entry protein	M. avium subsp. paratuberculosis K-10	100
MAP_2194	mammalian cell entry protein	M. avium subsp. paratuberculosis K-10	99
	hypothetical protein	M. avium subsp. paratuberculosis	100
MAP_2158	hypothetical protein	M. avium subsp. paratuberculosis K-10	100

	hypothetical protein	M. avium subsp. paratuberculosis	99
	hypothetical protein	M. avium subsp. paratuberculosis	99
	hypothetical protein	M. avium subsp. paratuberculosis	100
MAP_3765	PPE family protein	M. avium subsp. paratuberculosis K-10	100
MAP_3764c	polyketide synthase Pks2	M. avium subsp. paratuberculosis K-10	99
MAP_3763c	acyltransferase	M. avium subsp. paratuberculosis K-10	100
MAP_3762c	glycosyl transferase, UDP-glucuronosyltransferase	M. avium subsp. paratuberculosis K-10	100
MAP_3761c	hypothetical protein	M. avium subsp. paratuberculosis K-10	100
MAP_3760c	preprotein translocase subunit TatB	M. avium subsp. paratuberculosis K-10	98
MAP_0283c	hypothetical protein	M. avium subsp. paratuberculosis K-10	100
	hypothetical protein	M. avium subsp. paratuberculosis	99
MAP_2196	dihydrodipicolinate reductase	M. avium subsp. paratuberculosis K-10	100
MAP_2195	hypothetical protein	M. avium subsp. paratuberculosis K-10	100
MAP_0106c	putative phage integrase	M. avium subsp. paratuberculosis K-10	100
	XRE family transcriptional regulator	M. avium subsp. paratuberculosis	100
MAP_0105c	hypothetical protein	M. avium subsp. paratuberculosis K-10	100
MAP_2149c	hypothetical protein	M. avium subsp. paratuberculosis K-10	100
MAP_2148	recombinase XerD	M. avium subsp. paratuberculosis K-10	99
MAP_3815	hypothetical protein	M. avium subsp. paratuberculosis K-10	100
	hypothetical protein	M. avium subsp. paratuberculosis	98
MAP_3817c	membrane protein	M. avium subsp. paratuberculosis K-10	100
MAP_3818	cytochrome P450 hydroxylase	M. avium subsp. paratuberculosis K-10	100
MAP_3078c	transposase	M. avium subsp. paratuberculosis K-10	100

Locus 3

	type 11 methyltransferase	*M. avium subsp. paratuberculosis 11-1786*	100
	UDP-glucose 6-dehydrogenase	M. avium	99
	integrase	M. avium	100
	transposase	M. avium	100
	transposase	M. avium	100
	hypothetical protein	M. avium	100
	hypothetical protein	M. avium	99
	hypothetical protein	M. avium	100
	hypothetical protein	M. avium	100
	hypothetical protein	M. avium	99
	hypothetical protein	M. avium	99
	hypothetical protein	M. avium	98
	hypothetical protein	M. avium	100
	hypothetical protein	M. avium	100
	phage capsid protein	M. avium	100
	hypothetical protein	M. avium	99
	hypothetical protein	M. avium	100
	hypothetical protein	M. avium	100
	hypothetical protein	M. avium	100
	hypothetical protein	M. avium	100
	hypothetical protein	M. avium	100
	DNA-binding protein	M. avium	100
	integrase	M. avium	100
	NAD(+)--arginine ADP-ribosyltransferase Mav	*M. avium subsp. avium 11-4751*	100
	hypothetical protein	*M. intracellulare*	98
	16S rRNA (uracil(1498)-N(3))-methyltransferase	M. avium	100
	CoA transferase	M. avium	100
	maoC like domain protein	*M. avium subsp. avium 2285 (S)*	92
	amidohydrolase	M. avium	100
	isochorismatase hydrolase	*M. avium subsp. avium 10-9275*	100
	snoaL-like domain protein	*M. avium subsp. avium 10-9275*	100
	luciferase	*M. avium subsp. avium 10-9275*	99
	acyl-CoA dehydrogenase	*M. avium subsp. avium 10-9275*	99
	amidohydrolase	M. avium	99
	acyl-CoA dehydrogenase	*M. avium subsp. avium 11-4751*	100
	acyl-CoA dehydrogenase	M. avium	100
	cyclohexanecarboxylate-CoA ligase	M. avium	100
	amidohydrolase	M. avium	100
	amidohydrolase	*M. avium subsp. avium 11-4751*	99
	short-chain dehydrogenase	M. avium	100
	ABC transporter permease	M. avium	99
	hypothetical protein	*M. intracellulare*	100
	membrane protein	*M. avium subsp. avium 11-4751*	100
	membrane protein	*M. avium subsp. avium 11-4751*	95
	hypothetical protein	*M. avium subsp. avium 11-4751*	100
	AMP-dependent synthetase	M. avium	99
	enoyl-CoA hydratase	M. avium	100
	enoyl-CoA hydratase/isomerase	*M. avium subsp. avium 10-9275*	100
	acyl-CoA dehydrogenase	M. avium	100
	enoyl-CoA hydratase	*M. avium subsp. avium 10-9275*	100
	dehydratase MaoC	*M. avium subsp. avium 10-9275*	100
	enoyl-CoA hydratase	*M. avium subsp. avium 10-9275*	100
	short-chain dehydrogenase	*M. avium subsp. avium 10-9275*	100
	AMP-dependent synthetase	M. avium	100
	hypothetical protein	*M. avium subsp. avium 10-9275*	100
	thiolase	M. avium	100
	alpha-dehydro-beta-deoxy-D-glucarate aldolase	*M. avium subsp. avium 10-9275*	100
	gamma-butyrobetaine	M. avium	100
	3-phosphoglycerate dehydrogenase	*Mycobacterium avium complex (MAC)*	98
	3-phosphoglycerate dehydrogenase	*M. avium subsp. avium 11-4751*	100

crotonase	M. avium	99
amidohydrolase	M. avium	100
TetR family transcriptional regulator	*M. avium subsp. avium 10-9275*	100
acyl-CoA dehydrogenase	*Mycobacterium avium complex (MAC)*	100
acyl-CoA dehydrogenase	M. avium	99
phenylacetic acid degradation protein	*M. avium subsp. avium 10-9275*	100
enoyl-CoA hydratase	*M. avium subsp. avium 10-9275*	99
dihydrodipicolinate reductase	M. avium	99
nitroreductase	*M. avium subsp. avium 10-9275*	100
cytochrome P450	*M. avium subsp. avium 11-4751*	100
cytochrome P451	*M. avium subsp. avium 11-4751*	100
transcriptional regulator	*M. avium subsp. avium 10-9275*	99
cation-binding protein	*M. avium subsp. avium 10-9275*	100
ferredoxin	*M. avium subsp. avium 10-9275*	100
hemerythrin	*Mycobacterium avium complex (MAC)*	100
osmotically inducible protein OsmC	*M. avium subsp. avium 10-9275*	100
hypothetical protein	*M. avium subsp. avium 10-9275*	100
NAD(P)H nitroreductase	*M. avium subsp. avium 10-9275*	100
transcriptional regulator	M. avium	99
cyanoglobin	M. avium	100
(4Fe-4S)-binding protein	M. avium	100
membrane protein	M. avium	100
hypothetical protein	*M. intracellulare ATCC 13950*	100
transcription elongation factor GreA	*M. intracellulare ATCC 13950*	100
amino acid dehydrogenase	M. avium	100
membrane protein	M. avium	100
PucR family transcriptional regulator	*Mycobacterium avium complex (MAC)*	100
ferredoxin	*M. avium subsp. avium 10-9275*	100
SPFH domain-containing protein/band 7 family protein	*Mycobacterium avium complex (MAC)*	100
hypothetical protein	*M. intracellulare ATCC 13950*	100
hemerythrin	*M. avium subsp. avium 10-9275*	100
PPE11	M. avium	100
hypothetical protein	M. avium	100
hypothetical protein	M. avium	100
ABC transporter	*Mycobacterium avium complex (MAC)*	100
hypothetical protein	M. avium	100
malate dehydrogenase	M. avium	99
FAD-binding protein	M. avium	100
iron reductase	M. avium	100
Pantothenate synthetase	*M. avium subsp. avium 10-9275*	100
TetR family transcriptional regulator	*M. avium subsp. avium 10-9275*	100
hypothetical protein	*M. avium subsp. avium 10-9275*	100
short-chain dehydrogenase	*M. avium subsp. avium 10-9275*	100
LysR family transcriptional regulator	*M. avium subsp. avium 10-9275*	100
hypothetical protein	*M. avium subsp. avium 10-9275*	99
hypothetical protein	M. avium	100
hypothetical protein	M. avium	100
alcohol dehydrogenase	*M. avium subsp. avium 10-9275*	100
short-chain dehydrogenase	*M. avium subsp. avium 10-9275*	100
amidohydrolase	*M. avium subsp. avium 10-9275*	100
TetR family transcriptional regulator	*M. avium subsp. avium 10-9275*	100
dihydrodipicolinate reductase	*M. avium subsp. avium 10-9275*	100
dihydrodipicolinate reductase	*Mycobacterium avium complex (MAC)*	100
amino acid ABC transporter substrate-binding protein	*M. avium subsp. avium 10-9275*	100
dihydrodipicolinate synthase	*M. avium subsp. avium 10-9275*	99
cytochrome P450	*Mycobacterium avium complex (MAC)*	99
dihydrodipicolinate reductase, family protein	*Mycobacterium avium complex (MAC)*	100
hypothetical protein	*M. avium subsp. avium 10-9275*	100
short-chain dehydrogenase	*M. avium subsp. avium 10-9275*	100
clavaldehyde dehydrogenase	*M. avium subsp. avium 10-9275*	100
cytochrome P450	*M. avium subsp. avium 10-9275*	99
hypothetical protein	M. avium	100
dihydrodipicolinate reductase	*M. avium subsp. avium 10-9275*	99
alpha/beta hydrolase	*Mycobacterium avium complex (MAC)*	100
diacylglycerol kinase	*M. avium subsp. avium 10-9275*	100
oxidoreductase	*M. avium subsp. avium 10-9275*	100
hypothetical protein	*M. avium subsp. avium 10-9275*	100
AMP-dependent synthetase and ligase	*M. avium subsp. avium 10-9275*	100
polyketide cyclase	*M. avium subsp. avium 10-9275*	100
CoA transferase	M. avium	100
cytochrome P450	*M. avium subsp. avium 10-9275*	100
hypothetical protein	*M. avium subsp. avium 10-9275*	98
threonine dehydrogenase	*M. avium subsp. avium 10-9275*	100
hypothetical protein	*M. avium subsp. avium 10-9275*	100
xylose isomerase	*M. avium subsp. avium 10-9275*	100
short-chain dehydrogenase	*M. avium subsp. avium 10-9275*	100
sulfotransferase	*M. avium subsp. avium 10-9275*	100
oxidoreductase	*M. avium subsp. avium 10-9275*	100
MBL fold metallo-hydrolase	*Mycobacterium avium complex (MAC)*	100
transcriptional regulator	*M. avium subsp. avium 10-9275*	99
TetR family transcriptional regulator	*M. avium subsp. avium 10-9275*	100
lipid-transfer protein	*M. avium subsp. avium 10-9275*	100
acyl dehydratase	*M. avium subsp. avium 10-9275*	100
acyl-CoA dehydrogenase	*M. avium subsp. avium 10-9275*	100
acyl dehydratase	*M. avium subsp. avium 10-9275*	100
dehydratase MaoC	*M. avium subsp. avium 10-9275*	100
acyl-CoA dehydrogenase	M. avium	100

	cytochrome P450	M. avium	99
	aldehyde dehydrogenase	M. avium	99
	CoA transferase	M. avium	100
	hypothetical protein	M. avium	99
	hypothetical protein	*M. avium subsp. avium 10-9275*	97
	integrase	M. avium	100
	hypothetical protein	*M. brisbanense*	50
	hypothetical protein	M. avium	100
	hypothetical protein	M. avium	100
	terminase	M. avium	100
	hypothetical protein	M. avium	100
	hypothetical protein	*M. avium subsp. avium 10-9275*	100
	hypothetical protein	M. avium	100
	phage capsid protein	M. avium	100
	hypothetical protein	M. avium	100
	hypothetical protein	M. avium	100
	hypothetical protein	M. avium	100
	acetyltransferase	M. avium	100
	LLM class F420-dependent oxidoreductase	M. avium	100
	hemerythrin	M. avium	100
	manganese catalase	M. avium	99

Locus 4

MAH_2061	linear gramicidin synthetase subunit D	M. avium subsp. hominissuis TH135	100
MAH_2062	linear gramicidin synthetase subunit B	M. avium subsp. hominissuis TH135	100
MAH_2063	peptide synthetase NRP	M. avium subsp. hominissuis TH135	100
MAH_0292	hypothetical protein	M. avium subsp. hominissuis TH135	99
MAH_0303	F420-dependent methylene-tetrahydromethanopterin reductase	M. avium subsp. hominissuis TH135	100
MAH_0304	GntR family transcriptional regulator	M. avium subsp. hominissuis TH135	99
MAH_0305	nitrilotriacetate monooxygenase component A	M. avium subsp. hominissuis TH135	100
MAH_1620	hypothetical protein	M. avium subsp. hominissuis TH135	99
MAH_1621	ABC transporter	M. avium subsp. hominissuis TH135	100
MAH_1625	phospho-2-dehydro-3-deoxyheptonate aldolase	M. avium subsp. hominissuis TH135	100
MAH_2032	sulfotransferase family protein	M. avium subsp. hominissuis TH135	100
MAH_2033	NAD dependent epimerase/dehydratase	M. avium subsp. hominissuis TH135	100
MAH_2035	macrocin-O-methyltransferase	M. avium subsp. hominissuis TH135	100
MAH_2036	glycosyl transferase family 1	M. avium subsp. hominissuis TH135	100
MAH_2037	glycosyl transferase family protein	M. avium subsp. hominissuis TH135	100
MAH_2038	methyltransferase MtfC	M. avium subsp. hominissuis TH135	100
MAH_2039	methyltransferase	M. avium subsp. hominissuis TH135	100
MAH_2040	glycosyltransferase 28	M. avium subsp. hominissuis TH135	99
MAH_3546	putative oRFX protein	M. avium subsp. hominissuis TH135	100
MAH_3547	glutathione peroxidase	M. avium subsp. hominissuis TH135	100
MAH_3548	immunogenic protein MPT64	M. avium subsp. hominissuis TH135	100
	hypothetical protein	M. avium	100
MAH_3759	HTH-type transcriptional regulator	M. avium subsp. hominissuis TH135	99
MAH_3760	alpha-ketoglutarate-dependent taurine dioxygenase	M. avium subsp. hominissuis TH135	100
MAH_3761	cholesterol oxidase	M. avium subsp. hominissuis TH135	100
MAH_4286	PPE family protein	M. avium subsp. hominissuis TH135	100
	hypothetical protein	M. avium	100
MAH_4386	transmembrane protein	M. avium subsp. hominissuis TH135	100
MAH_4387	membrane protein	M. avium subsp. hominissuis TH135	100
MAH_4388	peptidase M13	M. avium subsp. hominissuis TH135	99
MAH_2194	erythronolide synthase, modules 3 and 4	M. avium subsp. hominissuis TH135	100
MAH_2238	linear gramicidin synthetase subunit D	M. avium subsp. hominissuis TH135	100
MAH_4549	bacterial regulatory, tetR family protein	M. avium subsp. hominissuis TH135	100
MAH_4550	hydrolase, alpha/beta hydrolase family protein	M. avium subsp. hominissuis TH135	99
MAH_4547	short chain dehydrogenase family protein	M. avium subsp. hominissuis TH135	100
MAH_4548	hypothetical protein	M. avium subsp. hominissuis TH135	100
MAH_4550	hydrolase, alpha/beta hydrolase family protein	M. avium subsp. hominissuis TH135	100
MAH_4551	methyltransferase type 11	M. avium subsp. hominissuis TH135	100
MAH_4552	polyketide cyclase	M. avium subsp. hominissuis TH135	100
MAH_4553	SAM-dependent methyltransferase	M. avium subsp. hominissuis TH135	97
MAH_4554	hypothetical protein	M. avium subsp. hominissuis TH135	100
MAH_4555	3-alpha-hydroxysteroid dehydrogenase	M. avium subsp. hominissuis TH135	100
MAH_4556	hypothetical protein	M. avium subsp. hominissuis TH135	100
MAH_4557	transcriptional regulator	M. avium subsp. hominissuis TH135	98
MAH_4558	snoaL-like domain protein	M. avium subsp. hominissuis TH135	100
MAH_4559	oxidoreductase, short chain dehydrogenase/reductase	M. avium subsp. hominissuis TH135	100
MAH_4560	sodium/calcium exchanger family protein	M. avium subsp. hominissuis TH135	100
MAH_4561	short chain dehydrogenase	M. avium subsp. hominissuis TH135	99
MAH_4562	monooxygenase	M. avium subsp. hominissuis TH135	100
	pyridoxamine 5'-phosphate oxidase	M. avium	100
MAH_4564	polyketide cyclase	M. avium subsp. hominissuis TH135	100

Locus 5

MAH_1592	TetR family transcriptional regulator	M. avium subsp. hominissuis TH135	100
MAH_1593	2,3-dihydroxybiphenyl-1,2-dioxygenase	M. avium subsp. hominissuis TH135	100
MAV_1975	hypothetical protein	*M. avium 104*	98
MAH_1603	short chain dehydrogenase	M. avium subsp. hominissuis TH135	100
MAH_1604	TetR family transcriptional regulator	M. avium subsp. hominissuis TH135	99
MAH_1605	dimethyl sulfoxide reductase	M. avium subsp. hominissuis TH135	100
MAV_1980	hypothetical protein	*M. avium 104*	100

MAH_1606	adenylate kinase	M. avium subsp. hominissuis TH135	100
MAV_1983	hypothetical protein	*M. avium 104*	100
MAH_1607	transcription elongation factor	M. avium subsp. hominissuis TH135	100
MAH_1608	transcription elongation factor GreA	M. avium subsp. hominissuis TH135	100
MAH_1609	transmembrane protein	M. avium subsp. hominissuis TH135	100
MAH_1610	DNA-binding protein	M. avium subsp. hominissuis TH135	100
MAH_1611	18 kDa antigen 2	M. avium subsp. hominissuis TH135	100
MAH_1613	hypothetical protein	M. avium subsp. hominissuis TH135	98
MAH_1614	HspR protein	M. avium subsp. hominissuis TH135	99
MAV_1994	hypothetical protein	*M. avium 104*	97
MAH_1615	diguanylate cyclase	M. avium subsp. hominissuis TH135	100
MAH_1616	ferredoxin	M. avium subsp. hominissuis TH135	100
MAV_1998	PPE family protein	*M. avium 104*	100
MAH_1619	hypothetical protein	M. avium subsp. hominissuis TH135	100
MAH_1620	hypothetical protein	M. avium subsp. hominissuis TH135	100
MAH_1621	ABC transporter	M. avium subsp. hominissuis TH135	100
MAH_1622	PapA2 protein	M. avium subsp. hominissuis TH135	100
MAH_1624	PPE family protein	M. avium subsp. hominissuis TH135	100
MAH_2296	oxidoreductase, molybdopterin binding	M. avium subsp. hominissuis TH135	100
MAH_2297	lipoprotein	M. avium subsp. hominissuis TH135	100
MAH_2298	adenylate-forming enzyme	M. avium subsp. hominissuis TH135	100
MAH_2299	class II aldolase	M. avium subsp. hominissuis TH135	100
MAH_2300	amidohydrolase	M. avium subsp. hominissuis TH135	100
MAH_2302	tetracenomycin polyketide synthesis O-methyltransferase TcmP	M. avium subsp. hominissuis TH135	99
MAH_2303	oxidoreductase	M. avium subsp. hominissuis TH135	100
MAH_2304	arylsulfatase	M. avium subsp. hominissuis TH135	100
MAH_2305	ubiquinone/menaquinone biosynthesis methyltransferase	M. avium subsp. hominissuis TH135	100
MAH_2307	putative export protein SecD	M. avium subsp. hominissuis TH135	100
MAH_2308	TetR family transcriptional regulator	M. avium subsp. hominissuis TH135	100
MAH_2309	cytochrome P450	M. avium subsp. hominissuis TH135	99
MAH_2310	carveol dehydrogenase	M. avium subsp. hominissuis TH135	100
MAH_2311	enoyl-CoA hydratase/isomerase	M. avium subsp. hominissuis TH135	100
MAH_2312	caib/baif family protein	M. avium subsp. hominissuis TH135	100
MAH_2313	clavaldehyde dehydrogenase	M. avium subsp. hominissuis TH135	100
MAH_2314	hypothetical protein	M. avium subsp. hominissuis TH135	100
MAH_2238	linear gramicidin synthetase subunit D	M. avium subsp. hominissuis TH135	100
MAH_2239	AsnB_1 protein	M. avium subsp. hominissuis TH135	100
MAH_2240	GNAT family acetyltransferase	M. avium subsp. hominissuis TH135	100
MAH_2241	sugar ABC transporter	M. avium subsp. hominissuis TH135	100
MAH_2242	putative transmembrane serine/threonine-protein kinase E	M. avium subsp. hominissuis TH135	100

Supplementary Table 3. Comparison of detection rates of Is*Mav6*, Is*Mav6* in *cfp29*, and each virulence factor between progressive and stable disease group.

	Progressive disease group (n = 17)	Stable disease group (n = 29)	*P* value[a]
Is*Mav6*[b] positive	10 (58.8%)	9 (31.0%)	0.12
Is*Mav6* in *cfp29*[b] positive	9 (52.9%)	3 (10.3%)	0.004
pMAH135[c] positive	6 (35.3%)	2 (6.9%)	0.038
mmpL family gene[d] positive	7 (41.2%)	3 (10.3%)	0.025
mce family gene[e] positive	7 (41.2%)	3 (10.3%)	0.025
mmpL family gene[f] positive	7 (41.2%)	3 (10.3%)	0.025

[a] *P* values were calculated using Fisher's exact test.
[b] Detection of Is*Mav6* and Is*Mav6* in *cfp29* was conducted by Nakagawa et al[51].
[c] pMAH135 was detected by the S1-PFGE analysis as shown in Fig. 5.
[d] *mmpL* family gene has sequence identity with MAH_0778 encoded by region SR-2 of strain TH135.
[e] *mce* family gene has sequence identity with MAH_0796 encoded by region SR-2 of strain TH135.
[f] *mmpL* family gene has sequence identity with CDS encoded by genome of *M. avium* 10-5581.
